# Functional organization of protein determinants of meiotic DNA break hotspots

**DOI:** 10.1038/s41598-017-00742-3

**Published:** 2017-05-03

**Authors:** Lijuan Ma, Kyle R. Fowler, Cristina Martín-Castellanos, Gerald R. Smith

**Affiliations:** 1 Division of Basic Sciences, Fred Hutchinson Cancer Research Center, Seattle, WA 98109 USA; 2 Instituto de Biología Funcional y Genómica, CSIC/University of Salamanca, C/Zacarías González 2, 37007 Salamanca, Spain; 3 Department of Microbiology and Immunology, University of California at San Francisco, San Francisco, CA 94143 USA

## Abstract

During *Schizosaccharomyces pombe* meiotic prophase, homologous chromosomes are co-aligned by linear elements (LinEs) analogous to the axial elements of the synaptonemal complex (SC) in other organisms. LinE proteins also promote the formation of meiotic DNA double-strand breaks (DSBs), the precursors of cross-overs. Rec10 is required for essentially all DSBs and recombination, and three others (Rec25, Rec27, and Mug20) are protein determinants of DSB hotspots – they bind DSB hotspots with high specificity and are required for DSB formation there. These four LinE proteins co-localize in the nucleus in an interdependent way, suggesting they form a complex. We used random mutagenesis to uncover recombination-deficient missense mutants with novel properties. Some missense mutations changed essential residues conserved among *Schizosaccharomyces* species. DSB formation, gene conversion, and crossing-over were coordinately reduced in the mutants tested. Based on our mutant analysis, we revised the *rec27* open reading frame: the new start codon is in the previously annotated first intron. Genetic and fluorescence-microscopy assays indicated that the Rec10 N- and C-terminal regions have complex interactions with Rec25. These mutants are a valuable resource to elucidate further how LinE proteins and the related SCs of other species regulate meiotic DSB formation to form crossovers crucial for meiosis.

## Introduction

Meiosis is a special process in which diploid cells undergo one round of DNA replication followed by two consecutive rounds of chromosome segregation to generate haploid gametes. In the first meiotic division, homologous chromosomes physically associate with each other via crossing-over during prophase, resulting in the reciprocal exchange of chromosome portions, and then segregate. This unique feature of meiosis – high-levels of homologous recombination – is critical for sexually reproducing species and is regulated in multiple ways. Homologous recombination is initiated by DNA double-strand breaks (DSBs) formed by the conserved protein Spo11 (called Rec12 in fission yeast, studied here)^1^. These programmed DSBs are not evenly distributed throughout the genome: the frequency of breakage is much higher than average at widely-spaced loci called DSB hotspots. Here, we describe novel mutants of proteins critical for determining DSB hotspots. These proteins also comprise a structure important for the pairing of homologs described below.

During meiotic prophase in most eukaryotes studied, a tripartite chromosomal structure called the synaptonemal complex (SC) is observed under the electron microscope [reviewed in ref. 2]. The SC consists of a central element and two parallel lateral elements to which chromatin loops are attached, thereby aligning and holding together the two homologous chromosomes. Axial elements, formed shortly after replication, become the lateral elements of the SC later in meiosis. The ultra-structure of the SC is highly conserved, but the proteins composing it are highly diverged among species.

In meiotic prophase of the fission yeast *Schizosaccharomyces pombe*, linear elements (LinEs), but not full-length SC, are detected by electron microscopy (EM) of spread-out nuclear contents; LinEs appear similar to lateral elements of the SC of other species but do not extend end-to-end on the chromosomes in such EM analyses^3, 4^. Like the SC, LinE formation requires a meiosis-specific sister chromatid cohesin complex containing Rec8 and Rec11 subunits, and phosphorylation of one of them (Rec11) by a casein kinase 1 ortholog^5, 6^. Rec8 forms filamentous structures with the chromosomes aligned in parallel from one end to the other during the horsetail stage of meiosis as observed in live cells by super-resolution structured illumination microscopy (SIM)^7^. This observation reveals a structural similarity of *S. pombe* meiotic chromosomes and those of other species. The time-course of LinE development, like that of the SC, is coordinated with other meiotic prophase events – LinEs form after replication but before recombination is complete^3, 8, 9^. Although LinEs differ somewhat from the SC in structure or stability, both SC and LinE proteins are required for high levels of homolog pairing and meiotic recombination^10–14^.

Four *S. pombe* proteins have been identified as LinE components – Rec10, Rec25, Rec27 and Mug20. The morphology of immunostained Rec10 is similar to that of LinEs observed by EM, suggesting Rec10 is a primary structural component^4^. In the C-terminal truncation mutant *rec10-155*, LinEs are not detectable by EM^13, 15^. Rec25, Rec27 and Mug20 are small proteins (125–151 amino acids) predicted to form coiled-coil domains, which are also found in SC proteins. In *rec25Δ* or *rec27Δ* cells, no Rec10 immunostaining signal, and therefore no LinEs, can be detected by fluorescence microscopy^11^. In the absence of Mug20, LinEs fail to develop to full-length^12^ or to form distinct nuclear foci^16^. Furthermore, these four LinE components interdependently co-localize in meiotic nuclei of live cells; i.e., focus-formation of any one depends on each of the others tested^11, 16^. Rec10 physically interacts with the other three proteins, as detected by mass spectrometry of immuno-precipitates, and directly with Rec25, as detected in yeast two-hybrid assays^12, 17^. Thus, these four proteins appear to function as a physical complex (designated the LinE complex), although there is no direct biochemical evidence for such a complex. Two other proteins, Hop1 and Mek1, also localize to LinEs and their localization depends on Rec10^9^.

LinE proteins are crucial for meiotic recombination. Like elimination of Rec12 or any of its partner proteins, deletion of *rec10* abolishes meiotically induced homologous recombination in all intervals tested and DSB-formation genome-wide^16, 18, 19^. Rec10 interacts with Rec15, a Rec12 partner protein, in two-hybrid assays^20^ and appears to activate Rec12 for DSB formation. Rec25, Rec27 and Mug20 are meiotic DSB hotspot determinants – they bind specifically to nearly all hotspots and are essential for most hotspot DSBs^16^. In contrast to deletion of *rec10*, deletion of *rec25, rec27* or *mug20* reduces recombination frequencies strongly in some intervals (100-fold or more) but only modestly in others (as little as 2-fold)^11, 12, 21^. Thus, Rec10 acts in DSB formation genome-wide, whereas Rec25, Rec27, and Mug20 seem to function primarily or exclusively at DSB hotspots.

Although LinE proteins play a fundamental role in DSB formation, their mechanistic role is still unclear. Reduced recombination frequency could be a consequence of reduced DSB formation, but the available data do not exclude an additional function of LinE proteins in DSB repair after break formation. Some *rec10* non-deletion mutants (*rec10-109*, *rec10-144* and *rec10-155*) were used to study LinE development and function in recombination and homologous chromosome pairing^4, 13, 15, 22^, but no *rec25*, *rec27* and *mug20* alleles other than deletions have been reported, to our knowledge. Non-deletion mutants are especially useful to study multi-functional proteins, such as LinE proteins involved in DSB formation, homolog pairing and recombination. Here, we identified multiple missense and nonsense alleles of *rec25*, *rec27*, *mug20* and *rec10* and investigated recombination and DSB formation in these mutants. We revised the *rec27* open reading frame and the deduced sequence of the Rec27 protein. We also investigated Rec25-GFP focus-formation in multiple *rec10* missense mutants. Our results demonstrate the importance of certain conserved amino acids, lend further insight into LinE formation and function, and provide material useful for further investigations. Our study of LinE proteins provides a new perspective on the still-unclear relationship between the SC (axial elements), DSB formation, and recombination.

## Results

### A screen for novel LinE protein mutants deficient in meiotic recombination

**Figure 1:**
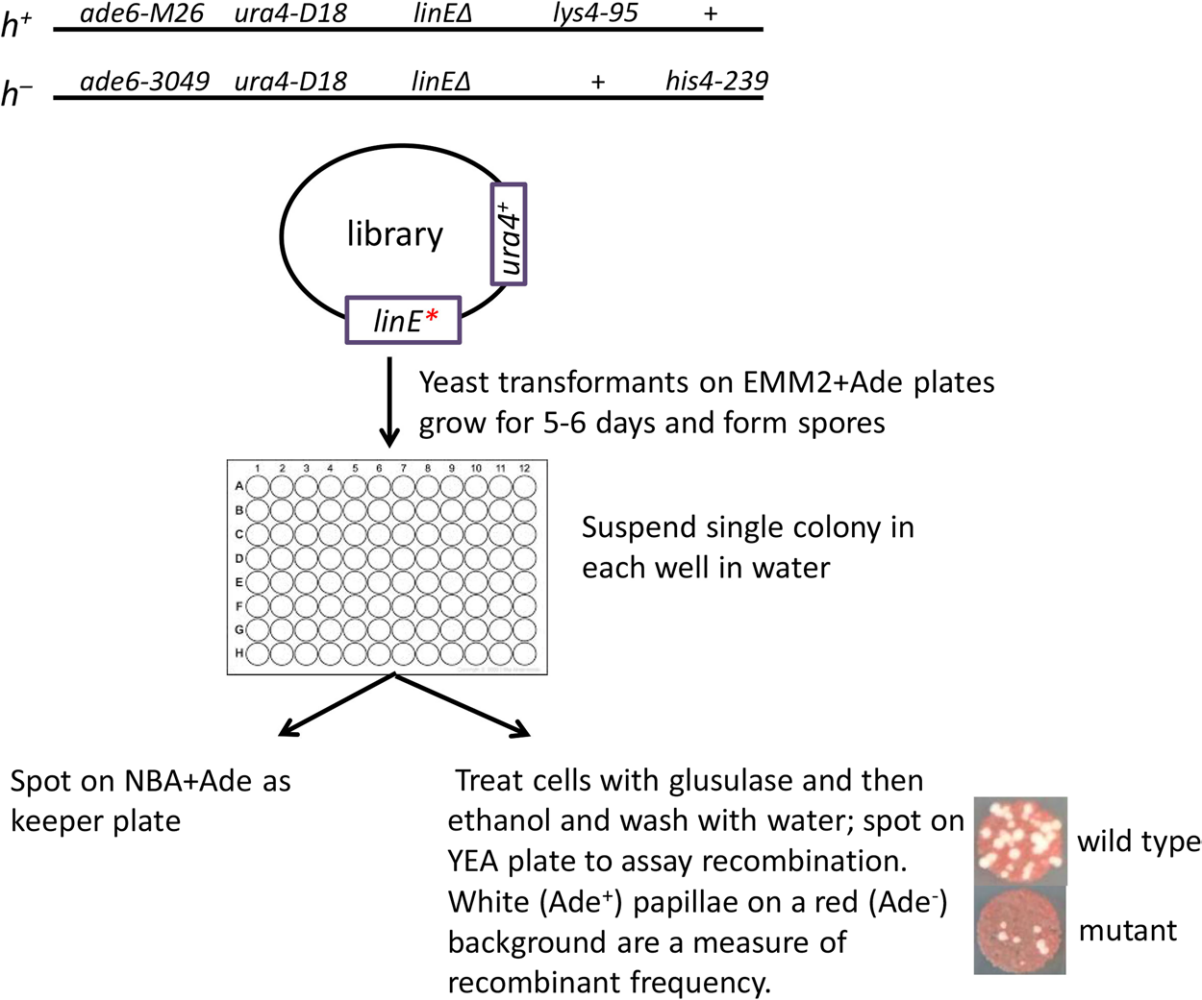
Schematic of the screen to isolate LinE mutants. A library of plasmids containing a randomly (PCR) mutagenized *rec25*, *rec27* or *mug20* gene was transferred to diploid cells homozygous for *rec25Δ*, *rec27Δ* or *mug20Δ*; linE* means *rec25*, *rec27* or *mug20*. After sporulation on EMM2+Ade plates, Ade^+^ recombinant frequencies were estimated by spot test (bottom right), in which recombination-deficient mutants produce a low frequency of white (Ade^+^) papillae on a background of red (Ade^−^) cells.

We designed a genetic screen to isolate new *rec25*, *rec27*, and *mug20* mutations affecting meiotic recombination by modifying a screen for recombination-deficient (Rec^−^) mutants described previously^23^ (Fig. 1). We used a diploid strain heteroallelic for *mat* (*h* ^+^/*h* ^−^) and for two non-complementing *ade6* mutations (*ade6-3049*/*ade6-M26*). Upon starvation, these cells undergo meiosis and recombine to *ade* ^+^ at high frequency due to the *ade6-3049* and *ade6-M26* recombination hotspots^19^. *ade6-3049* and *ade6-M26* are also DSB hotspots; each is a single base-pair change mutation of *ade6* ^+^ that creates a binding site for the transcription factor Atf1-Pcr1^24, 25^. Binding of Atf1-Pcr1, as well as LinE proteins, is required for DSB formation there^16, 26^. Chromatin structural changes accompanying this binding appear to facilitate, by an unknown mechanism, DSB formation by Rec12 and its putative partner proteins^27–29^. A LinE gene, such as *rec25*, was deleted on each chromosome but was present on a derivative of the low copy plasmid pFY20^30^ as a mutagenized library. After sporulation, spores were spotted on YEA plates without supplementary adenine, on which non-recombinant (Ade^−^) spores form red colonies, while recombinant (Ade^+^) spores form white colonies; this produces a red lawn with some white (Ade^+^) papillae. When the diploid cells express a recombination-deficient (Rec^−^) LinE mutant protein from the plasmid, the frequency of white papillae is reduced.

We used mutagenic PCR to form a library of plasmids with mutations restricted to the LinE protein gene. We sequenced the LinE protein gene from randomly chosen plasmids (9 for *rec25*, 10 for *rec27*, and 8 for *mug20*) and found 0.27 base-pair changes per *rec25* gene (0.12/kb), 2.5 base-pair changes per *rec27* gene (1.2/kb), and 0.65 base-pair changes per *mug20* gene (0.36/kb). We transformed an appropriate diploid strain with these libraries and screened ~400 transformants for *rec25* or *rec27* and ~200 transformants for *mug20*. We identified 43, 32, and 15 Rec^−^ candidates (*i.e*., with reduced frequency of white papillae) of *rec25, rec27*, and *mug20*, respectively. Among these candidates, 24, 17, and 6 were sequenced (Suppl. Figs S1–S3). Missense mutants, especially those changed in conserved amino acids, were chosen for further study. For all subsequent experiments reported here, the mutations were put onto the chromosome at the endogenous location, to ensure stability and appropriate gene expression.

### Four amino acids essential for Rec25 activity

**Figure 2:**
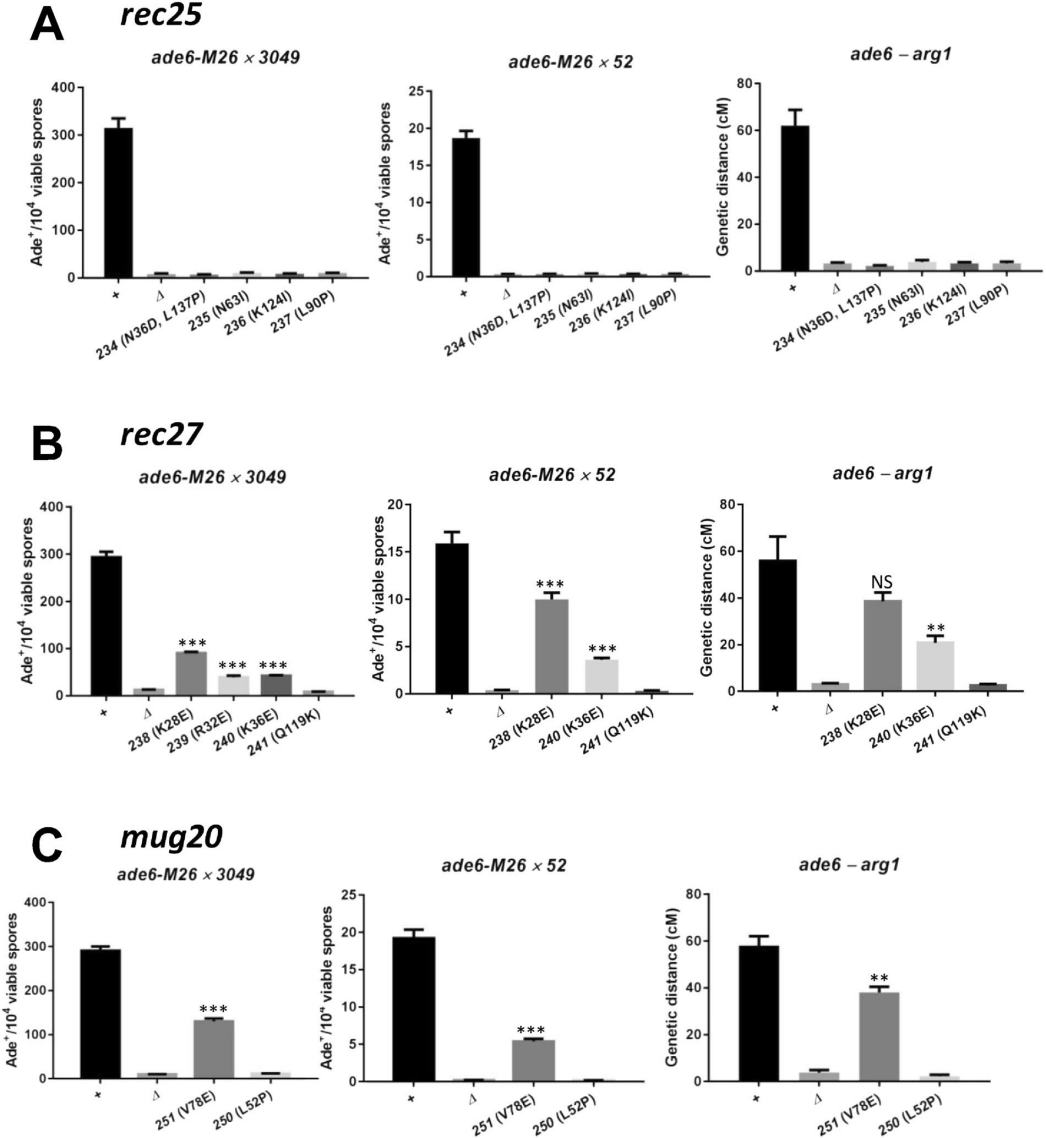
Meiotic recombination of newly isolated LinE mutants. Meiotic gene conversion at *ade6* (two allele pairs) and crossing-over between *ade6* and *arg1* in LinE mutants were measured as described in Methods. Data (mean ± SEM) are from Supplementary Table S3; n ≥ 3 for each mutant. NS (not significant; P > 0.05), **(P < 0.01), and ***(P < 0.001) indicate significance of the difference from wild type by unpaired t-test.

We analyzed four missense mutations of *rec25* – *rec25-234* (N36D, L137P), *rec25-235* (N63I), *rec25-236* (K124I), and *rec25-237* (L90P) – with substitutions of amino acids conserved in at least four of the five examined *Schizosaccharomyces* species (N36 is not conserved; Suppl. Fig. S4A). Each of these mutations reduced recombinant frequencies, both *ade6* intragenic (gene conversion) and *ade6 – arg1* intergenic (crossing-over), by factors not significantly different from that of the *rec25* deletion (null) mutation (Fig. 2A; Suppl. Table S3; by unpaired t-test p > 0.07, except for *rec25-235* gene conversion in the *ade6-M26* x *ade6-52* cross with p = 0.04). *ade6* gene conversion was reduced by factors of 30–50, and *ade6 – arg1* crossing-over by factors of 16–28, indicating the importance of Rec25, and these residues specifically, for recombination.

### Revised *rec27* ORF starts within the annotated first intron

**Figure 3:**
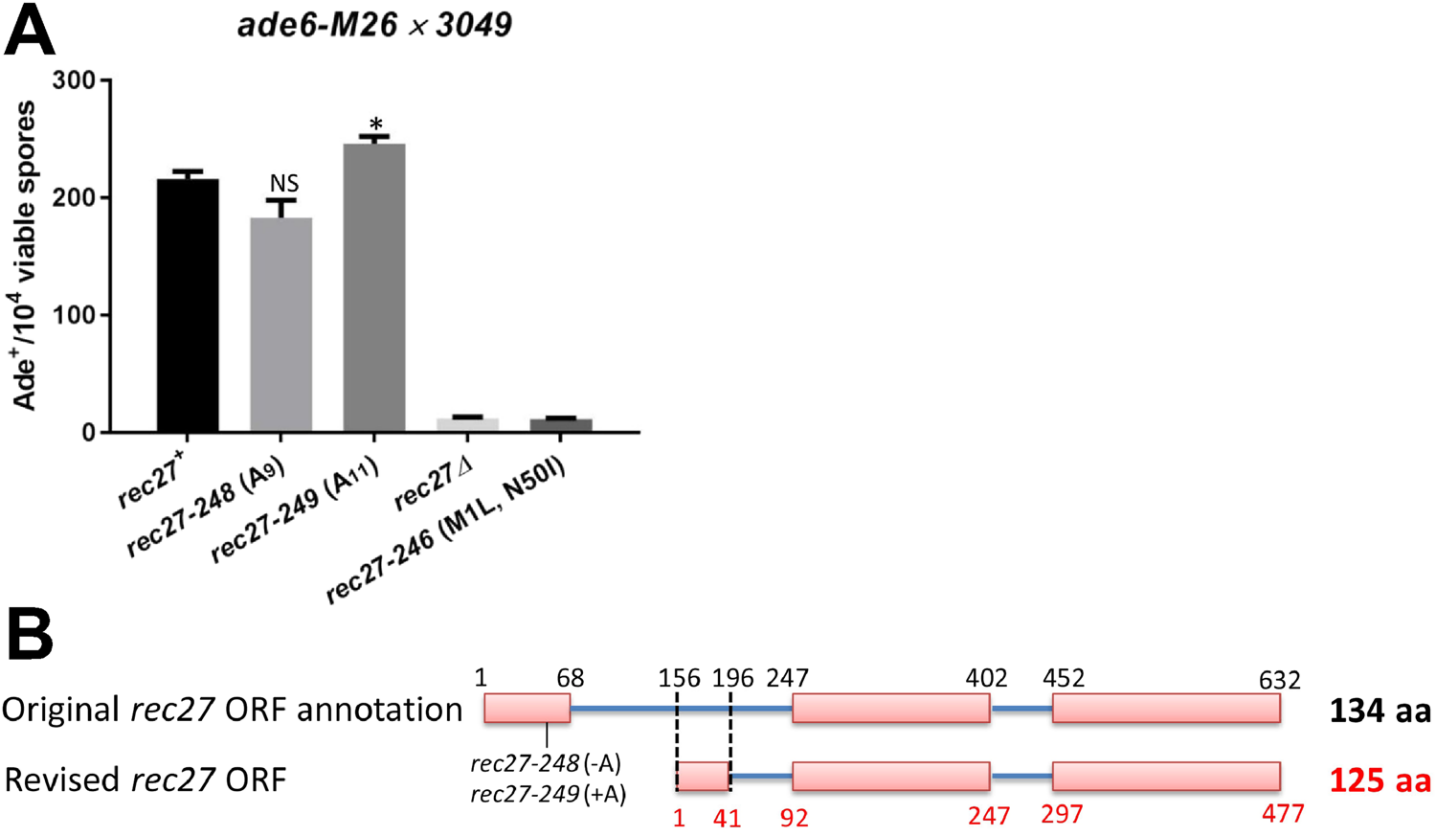
Revised Rec27 ORF starts within the first formerly designated intron. (**A**) Meiotic recombination between *ade6-M26* and *ade6-52* in *rec27* mutants. Data (mean ± SEM) are from Supplementary Table S3; n = 4 for each strain. NS (non-significant) and * (P < 0.05) indicate significance of the difference from *rec27* ^+^ by unpaired t-test. (**B**) Map of revised *rec27* ORF (lower line) compared to the previous annotation (upper line) (http://www.pombase.org/spombe/result/SPBC577.05c). Pink boxes are exons.

The *rec27* open reading frame (ORF) in the public database (http://www.pombase.org/) has ten sequential As (adenine) in the first exon close to the designated ATG start codon. We sequenced the *rec27* ^+^ ORF of two not-closely-related wild-type strains (GP13 and GP20) and found that each had ten sequential As, as expected. We found, however, that two isolates from our screen had only nine As (*rec27-248*) or eleven As (*rec27-249*) but were as recombination-proficient (Rec^+^) as another isolate with ten As, which we designate wild type (*rec27* ^+^) (Fig. 3A). These potential frameshift mutations would result in truncated Rec27 proteins only 20 and 22 amino acids long, yet they had wild-type recombination phenotype.

This unexpected observation raises the question, where does *rec27* translation start? Recent RNA-seq data show abundant transcripts covering only part of the former first intron, followed by a short exon and then the rest of the gene as designated in the database^31^. We fortunately recovered a mutation in the first annotated intron [*rec27-246* (M1L, N50I)] which exhibited the mutant null phenotype and changed a Met codon to a Leu codon. This Met codon, but not another Met codon toward the 5′ end of this intron, is covered by the RNA-seq data. We conclude, based on these data, that *rec27-246* changed the true start codon ATG (Met) to TTG (Leu) in what we conclude is the wild-type first exon (Table 1 and Fig. 3). Using these data, we revised the *rec27* ORF such that *rec27* translation starts within the former first intron at nucleotide (nt) 156 (relative to the previously designated ATG). Thus, the newly designated first exon extends from nt 156 to 196 and the new first intron extends from nt 197 to 247 (Fig. 3B). As a result, the predicted Rec27 protein has nine amino acids less than the annotated Rec27 protein and 12 amino acids different at the N-terminus. As expected, the revised Rec27 protein aligned better with other *Schizosaccharomyces* species Rec27 and *C. elegans* SYP-2 proteins, which were previously shown to share sequence similarity (Fowler *et al*.^16^; Suppl. Fig. S4B). The nucleotide changes and amino acid changes of the *rec27* mutants listed in Table 1 and Suppl. Fig. S2 were defined based on the revised ORF.

**Table 1:** Nucleotide and amino acid changes in LinE mutants.

Allele number	Nucleotide change^a^	Amino acid change
*rec25-234*	A106G, T410C	N36D, L137P
*rec25-235*	A188T	N63I
*rec25-236*	A371T, C421A^b^	K124I
*rec25-237*	T269C	L90P
*rec27-238*	A132G	K28E
*rec27-239*	A144G, G145A	R32E
*rec27-240*	A156G	K36E
*rec27-241*	C454A^c^	Q119K
*rec27-242*	A204G^d^	K52E
*rec27-243*	A219G^d^	K57E
*rec27-244*	A243G^d^	K65E
*rec27-245*	A421G^d^	K108E
*rec27-246*	A1T, A199T	M1L^e^, N50I
*rec27-248*	A_9_ at −104 from ATG	None
*rec27-249*	A_11_ at −104 from ATG	None
*mug20-250*	G70A^b^, T198C	L52P
*mug20-251*	T276A	V78E
*rec10-109*	G526A, G533A	V176I, G178D
*rec10-116*	G1725A	W575*
*rec10-133*	C2185T	Q729*
*rec10-134*	G811A, G1301A	E271K, R434H
*rec10-136*	G925A, G1809A	E309K, W603*
*rec10-144*	G2180A^f^	G727E^f^
*rec10-155*	*LEU2* insertion after G2070^g^	E691I and frame-shift^g^
*rec10-216*	AGGGAT (550–555)TTCACC^h^	R184F, D185T

^a^Relative to ATG (A = bp 1 of the revised *rec27* ATG; see Fig. 3B).

^b^Nucleotide change does not change protein coding.

^c^With additional mutation A_9_ at −104 from ATG.

^d^Site-directed mutation.

^e^The nucleotide change at the translational start, from ATG to TTG, is unlikely to allow translation to begin.

^f^From^22^.

^g^From^11^.

^h^Isolated by random mutagenesis of two codons (R184 and D185).

^*^Non-sense mutation.

### Four *rec27* missense mutations with recombination-reduced or null phenotype

Three *rec27* missense mutants – *rec27-238* (K28E), *rec27-240* (K36E), and *rec27-241* (Q119K) – were modestly or strongly Rec^−^ in meiosis. The meiotic gene conversion at *ade6* was reduced only 2- to 7-fold in *rec27-238* and *rec27-240* compared to that in wild-type cells. Crossing-over in the *ade6 – arg1* interval was reduced by a factor of 1.4 for *rec27-238* and by a factor of 3 for *rec27-240* (Fig. 2B; Suppl. Table S3). *rec27-241*, however, was strongly Rec^−^: it reduced both gene conversion and crossover frequencies to *rec27Δ* levels (Fig. 2B; Suppl. Table S3). *rec27-238* and *rec27-240* mutations changed K (lysine) to E (glutamic acid) at conserved sites. Interestingly, the mutation with the greatest defect (Q119) is not among the conserved sites of *Schizosaccharomyces* species; it is the seventh amino acid from the C-terminus and is apparently missing in two other related species (Table 1; Suppl. Fig. S4B).

Since the Rec^−^ mutants *rec27-238* (K28E) and *rec27-240* (K36E) altered conserved K (lysine) to E (glutamic acid), we used site-directed mutagenesis to similarly alter five other conserved K or R (arginine) sites to E (R32E, K52E, K57E, K65E and K108E) (Table 1)*. rec27-239* (R32E), but not the other mutations – *rec27-242* (K52E), *rec27-243* (K57E), *rec27-244* (K65E) and *rec27-245* (K108E) – modestly reduced meiotic recombination (Fig. 2B; Suppl. Fig. S5; Suppl. Table S3). The amino acids altered in the three Rec^−^ mutations, K28E, R32E and K36E, are conserved and clustered, suggesting this region is part of a functional domain.

### Two *mug20* missense mutations with recombination-reduced or null phenotype

In two *mug20* missense mutants – *mug20-250* (L52P) and *mug20-251* (V78E) – gene conversion and crossing-over were reduced. Both types of recombination were modestly reduced in *mug20-251* (1.5 to 3-fold) but were reduced to the level of *mug20Δ* in *mug20-250* (Fig. 2C; Suppl. Table S3). The amino acids changed in these two mutants (V78 and L52) are not conserved in *Schizosaccharomyces* species other than the closest related species *S. kambucha* (Suppl. Fig. S4C).

### Analysis of eight new and previously isolated *rec10* mutations

Rec10 is essential for meiotic recombination: deletion of *rec10* decreases recombinant frequencies about 1000-fold, to the levels in mutants lacking Rec12, the protein with the active site for DSB formation (Table 2)^19^. Here we analyzed seven *rec10* mutants (*rec10-109*, *rec10-116*, *rec10-133*, *rec10-134*, *rec10-136*, *rec10-144* and *rec10-155*) previously described but not sequenced (except for *rec10-144* and *rec10-155*)^11, 19, 22, 23, 32, 33^ and one newly created mutant (*rec10-216*).

**Table 2:** Locus-specific reduction of meiotic recombination in *rec10* mutants.

Mutant	Gene conversion *ade6-M26* × *52* ^a^	Crossing-over (cM)^b^		
		*lys3 – ura1*	*ura1 – met5*	*ade6 – arg1*
*rec10* ^+^	1200 ± 41 (4)	14	28	59 ± 4 (12)^c^
*rec10Δ* ^d^	2	0.2^e^	0.5^e^	<0.4^f^
*rec10-109*	27	13	35	ND^g^
*rec10-116*	4.5	1.3	9.2	ND
*rec10-133*	13^h^	ND	ND	ND
*rec10-134*	8.1	3.9	13	ND
*rec10-136*	21^h^	ND	ND	ND
*rec10-144*	85	3.5	6.3	ND
*rec10-155*	3.8	5^e^	16^e^	ND
*rec10-216*	8.3 ± 0.5 (4)	ND	ND	1.2 ± 0.3 (4)
*rec25Δ* ^i^	41	3.4	9.6	ND
*rec10-109*/*rec10-144*	650 ± 20 (4)	13^j^	31^j^	ND
*rec10-109*/*rec10-155*	11 ± 0.8 (4)	8^j^	15^j^	ND
*rec10-216*/*rec10-144*	200 ± 21 (4)	ND	ND	ND
*rec10-216*/*rec10-155*	2 ± 0.25 (4)	ND	ND	ND
*rec10-109 rec25Δ*	2	2.6	5.2	ND
*rec10-155 rec25Δ*	8.7 ± 0.6 (4)	3^e^	11^e^	ND
*rec10-144 rec25Δ*	24 ± 2 (4)	3^j^	ND	ND

^a^Ade^+^ spores/million viable spores, as mean of two independent experiments or mean ± SEM of four independent experiments.>200 total colonies and >20 Ade^+^ colonies were counted for each determination except for *rec10Δ, rec10-116, rec10-109*/*rec10-155* and *rec10-109 rec25Δ* (less than 20 Ade^+^ colonies were counted).

^b^120 spore colonies from each cross were tested for recombinants. Frequencies were converted to cM using Haldane’s equation.

^c^Collective data for *rec25* ^+^, *rec27* ^+^ and *mug20* ^+^ (Supplementary Table S3).

^d^ *rec10-175::kanMX6*.

^e^From^11^; wild-type genetic distances were 15 cM (*lys3 – ura1*) and 31 cM (*ura1 – met5*).

^f^From^19^; wild-type genetic distance was 73 cM (*ade6 – arg1*).

^g^ND, not determined.

^h^From^32^; Ade^+^ recombinants were from homothallic *h* ^*90*^ *ade6-M26 ura4-294 rec10-133* (or *rec10-136*) crossed with heterothallic *h* ^*–*^ *ade6-52 rec10-109*; wild-type frequency was 1600.

^i^ *rec25-180::kanMX6*.

^j^About 280 spore colonies from 4 independent crosses were tested. Frequencies were converted to cM using Haldane’s equation.

The first *rec10* mutant isolated, *rec10-109*, had two nearby amino acids changed in its conserved N-terminal quarter – V176I and G178D (Suppl. Fig. S4D; Table 1). Gene conversion at *ade6* was reduced by a factor of 100, but, remarkably, crossing-over in the *lys3* – *ura1* or *ura1 – met5* intervals on another chromosome was not affected, compared to wild-type cells (Table 2). This observation is consistent with the previous results with *rec10-109* which indicated that Rec10 is a region-specific activator of meiotic recombination^15, 23, 33^.

*rec10-216* (R184F, D185T) was newly generated by random mutagenesis of two N-terminal codons for amino acids conserved in all *Schizosaccharomyces* species (Suppl. Fig. S4D) and close to the sites mutated in *rec10-109*. *rec10-216* reduced gene conversion and *ade6 – arg1* crossing-over nearly to the level of *rec10Δ* (Table 2). *rec10-116* (W575*), *rec10-133* (Q729*), *rec10-134* (E271K, R434H), and *rec10-136* (E309K, W603*) were previously isolated as nitrosoguanidine-induced Rec^−^ mutants^32^, and three contain non-sense mutations (designated by *). *rec10-155* is an insertion of *S. cerevisiae LEU2* into the coding sequence^34^ and results in Rec10 lacking the C-terminal 101 amino acids with ten additional amino acids encoded by the insertion^11^ (Suppl. Fig. S6). Two mutations – *rec10-116* (W575*) and *rec10-155* (E691I and frame-shift) – decreased *ade6* gene conversion to the level of *rec10Δ*. Crossovers, however, were decreased substantially less: < 11-fold reduction in these mutants but > 50-fold reduction in *rec10Δ* ^11, 19^ (Table 2). *rec10-144* (G727E) changes an amino acid in a conserved region that is removed by the *rec10-155* truncation (E691I and frame-shift). Both *rec10-144* and *rec10-155* reduced recombination similarly for crossing-over, but *rec10-144* had less effect on gene conversion at *ade6* (Table 2).

We conducted complementation analyses by testing N-terminal mutations *rec10-109* (V176I, G178D) and *rec10-216* (R184F, D185T) with the C-terminal truncation mutation *rec10-155* (E691I and frame-shift) and the missense mutation *rec10-144* (G727E). Both *rec10-109* and *rec10-216* partially complemented the missense mutation *rec10-144*, consistent with previous reports of *rec10-109* and *rec10-144* complementation^22, 32^, but failed to complement the truncation mutation *rec10-155* (Table 2). Deletion of *rec25* in the N-terminal mutant *rec10-109* reduced the recombinant frequency to the *rec10Δ* level at *ade6* and also significantly reduced crossing-over (p < 0.01 by chi-square test). But deletion of *rec25* in the C-terminal *rec10-155* truncation or even in the missense mutation *rec10-144* did not have this strong additive affect (Table 2). These results suggest that Rec25 interacts with the C-terminal region of Rec10 and agrees with the absence of LinEs in the *rec10-155* C-terminal truncation mutant^15^.

### DSBs are reduced or eliminated in parallel with recombination reduction in the newly isolated LinE mutants

**Figure 4:**
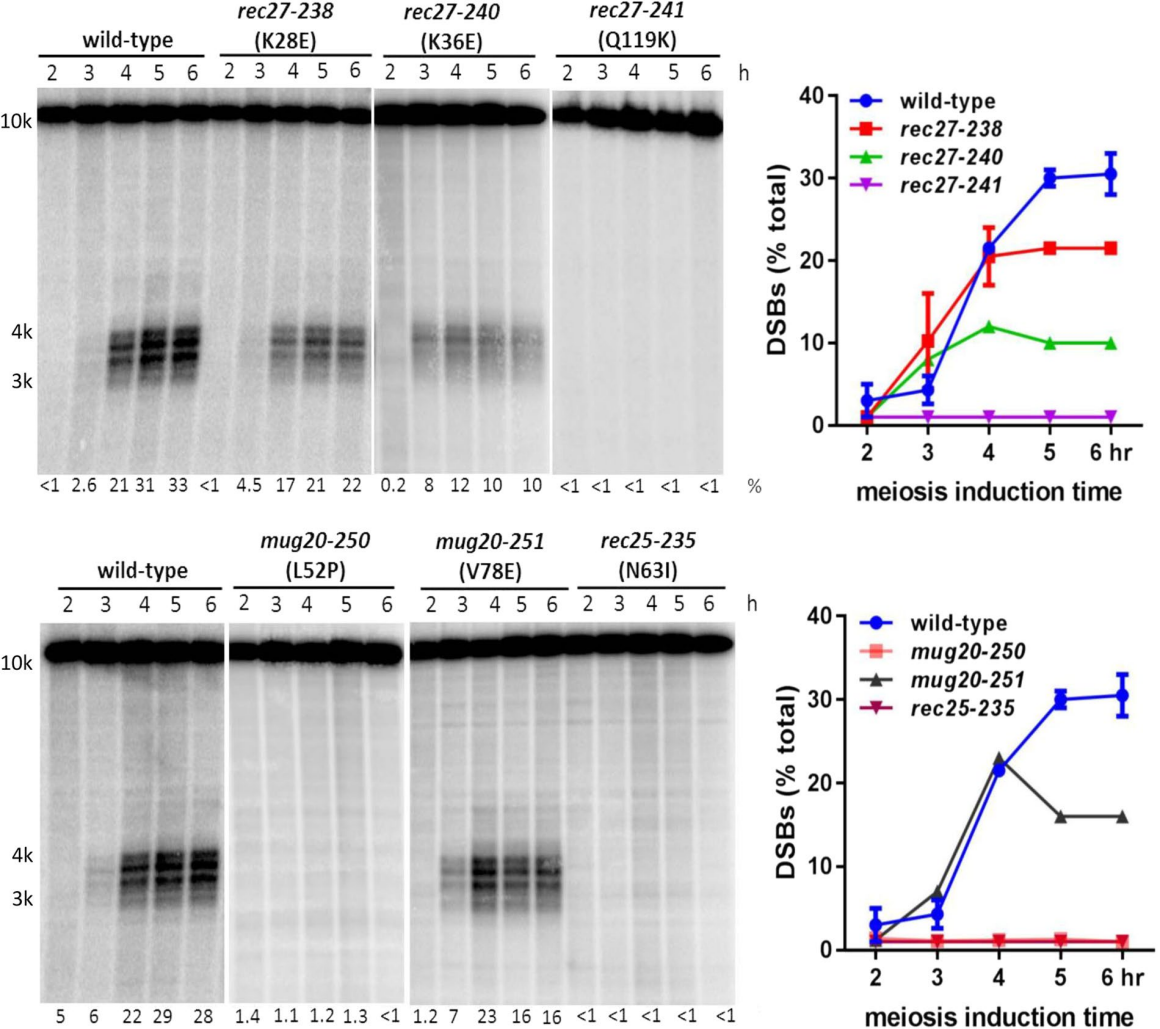
Meiotic DSBs of LinE mutants. LinE mutants were meiotically induced; DNA was extracted at the indicated times, digested with *Bsr*G1 and analyzed for DSBs at the *ade6-3049* hotspot by Southern blot hybridization. Unbroken DNA (10.1 kb) migrates at the top of the blots. Meiotically broken DNA (3–4 kb) migrates as multiple bands near the middle of the blots. The percentage of total radioactivity in the DSB position was quantified with ImageQuant and is shown below each lane and in the graph on the right. Data are individual values or (for wild-type and *rec27-238* cells) the range for two independent experiments. Full-length blots are presented in Suppl. Figure S7.

The deficiency of recombination observed in LinE mutants could result from failure either to form or to repair DSBs. DSBs are abolished genome-wide in *rec10Δ* mutants and strongly reduced at DSB hotspots in *rec27Δ*; at the few hotspots tested, DSBs are also strongly reduced in *rec25Δ* ^16, 19, 21^. We therefore assayed DSB formation at the *ade6-3049* hotspot in representative novel LinE mutants. Cells were induced to enter meiosis synchronously using the *pat1-114* temperature-sensitive repressor of meiosis in the *rad50S* background to accumulate unrepaired broken DNA^18^. There were no detectable DSBs in strong Rec^−^ mutants – *rec27-241* (Q119K), *rec25-235* (N63I) and *mug20-250* (L52P) (Figs 4 and S7). In weaker Rec^−^ mutants – *rec27-238* (K28E), *rec27-240* (K36E) and *mug20-251* (V78E) – DSBs at *ade6-3049* accumulated to 22%, 10% and 16%, respectively, of total DNA, compared to 30% in wild-type cells (mean percentage of broken DNA at 5 hr and 6 hr). Overall, DSBs were reduced in parallel with the reduction of recombinant frequencies (Figs 2 and 4).

### Rec25-GFP forms nuclear foci in a *rec10* C-terminus mutant but not in an N-terminus mutant

**Figure 5:**
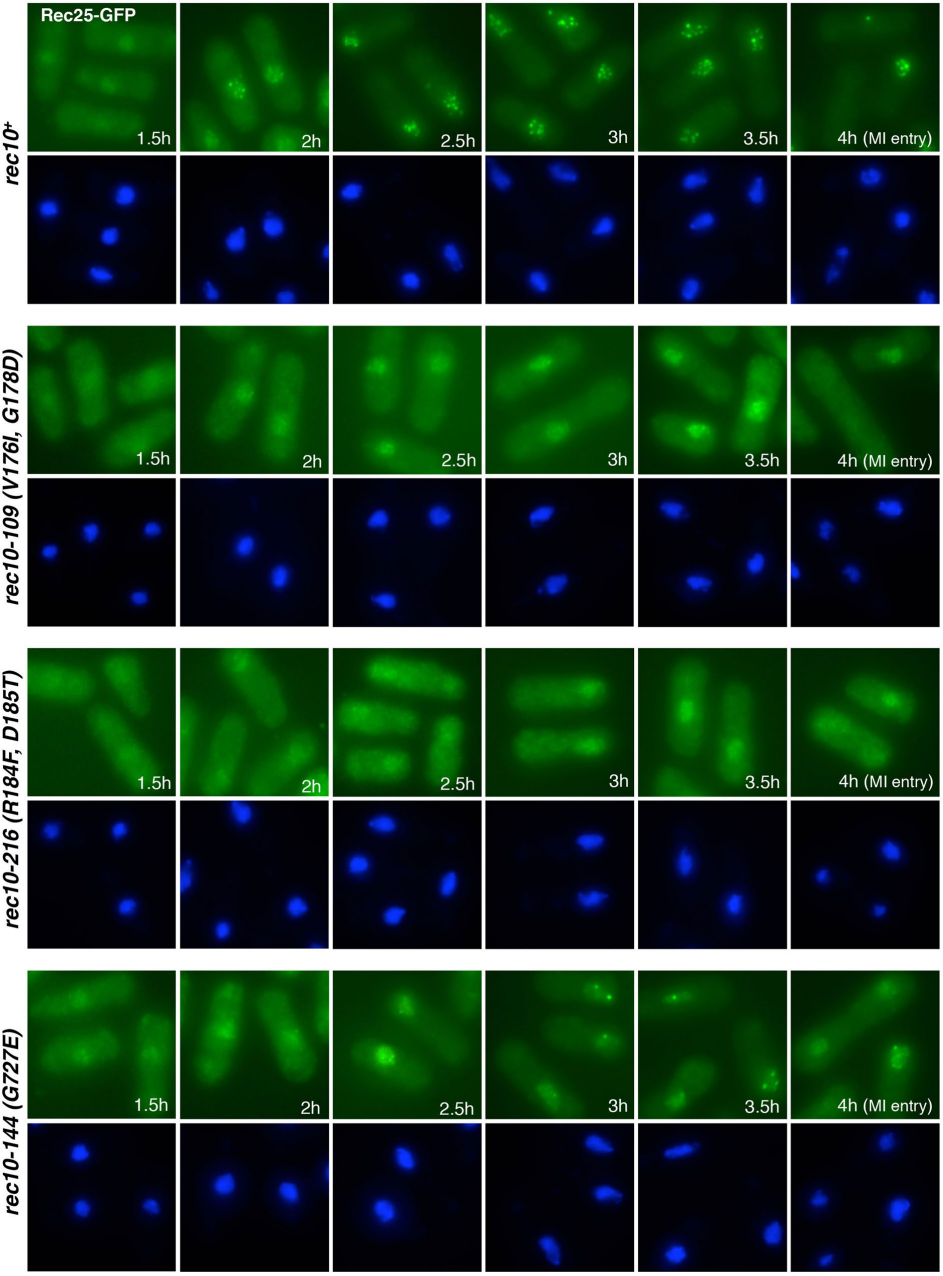
Rec25-GFP localization in *rec10* missense mutants. Cells containing Rec25-GFP and the indicated *rec10* mutations were induced for meiosis and fixed for fluorescence microscopy. The upper panels show Rec25-GFP, while the lower panels show DAPI-stained DNA. The figures show only the time points when GFP signal was detected (from 1.5 hr to 4 hr) after meiotic induction.

The formation of nuclear foci by Rec25-GFP, Rec27-GFP, Mug20-GFP and immunostained-Rec10 in nuclear spreads depends on the presence of each of the other tested LinE proteins, suggesting that these four proteins form an active complex^11, 12, 16^. To test whether mutations in the largest protein, Rec10, interrupt formation of this putative complex, we investigated Rec25-GFP nuclear focus-formation in three *rec10* mutants. *rec10-109* (V176I, G178D) and *rec10-216* (R184F, D185T) alter the N-terminal region, and *rec10-144* (G727E) alters the C-terminal region. In *rec10-109* cells, Rec25-GFP fluorescence became visible in the nucleus about 1.5 hr after meiotic induction, similarly as in wild-type cells, but the Rec25-GFP foci were scarce, faint or not distinct, even at 4 hr after meiotic induction (Fig. 5). In *rec10-216* cells, Rec25-GFP entered the nucleus with similar kinetics, but the signal was less concentrated (weaker, and without distinct foci) than in wild-type cells, especially at early time points, and nuclear accumulation was not clear until later in meiosis (Fig. 5). This suggests that in *rec10-216* mutant cells Rec25-GFP protein does not enter the nucleus efficiently. Flow cytometry showed that DNA replication in *rec10-109* and *rec10-216* cells was normal, indicating that the retarded Rec25-GFP accumulation and focus formation in the nucleus was not caused by delayed meiotic progression (data not shown); indeed, both mutants entered meiosis I with normal timing compared to control cells (Fig. 5). In *rec10-144* cells, Rec25-GFP appeared in the nucleus at the same time as in wild-type cells, and with similar intensity, but only a few foci were formed on top of a pan-nuclear signal not observed in the control at times when discrete Rec25-GFP foci were present (Fig. 5).

## Discussion

Missense mutations are especially useful to study a protein’s function, particularly that of multi-functional proteins such as the *S. pombe* LinE proteins. We report here eleven newly isolated missense mutations of LinE proteins – four *rec25* mutants, four *rec27* mutants, two *mug20* mutants and one *rec10* mutant – and their levels of meiotic recombination, DSB formation and putative complex formation to understand further how these proteins act in meiosis. Additionally, we revised the *rec27* ORF.

LinE components share some similarities with SC proteins found in other species. Rec10 has limited amino-acid sequence similarity to Red1, an *S. cerevisiae* axial element protein; the similarity is limited to ~10% of the protein near the C-terminus that in both proteins is predicted to form a coiled-coil^4^ (Suppl. Fig. S4D). Red1 appears to play a role in the bias toward DSB repair with the homolog rather than with the sister chromatid^35, 36^. Rec27 and Mug20 share amino-acid sequence similarity with *Caenorhabditis* SC proteins SYP-2 and DDL-1, respectively^16^. Rec25, Rec27, and Mug20 are also predicted to be coiled-coil proteins, a feature of many SC components from numerous organisms. In addition, LinE components are highly conserved among *Schizosaccharomyces* species. For example, the *S. pombe* and *S. kambucha* genomes share 99.5% nucleotide sequence identity^37^, and their Rec25, Rec27, and Mug20 proteins are 100% identical, and Rec10 98% identical (Suppl. Fig. S4). Among all annotated orthologs in the genomes of three other *Schizosaccharomyces* species, the mean amino-acid sequence identities, compared to those of *S. pombe*, range from 66% for *S. octosporus* to 55% for *S. japonicus* ^37^. Among the four individual LinE proteins, amino-acid sequence identities, compared to those of *S. pombe*, range from 40% for Mug20 of *S*. *cryophilus* to 25% for Rec10 of *S*. *japonicus*. Although these LinE proteins are somewhat less conserved than proteins in general, as is typical for meiotic proteins, the conclusions with *S. pombe* mutants, described here, are likely to extend to the other *Schizosaccharomyces* species and perhaps to more distant species such as *C. elegans* as well.

In the *rec25*, *rec27* and *mug20* mutants studied here, there is co-ordinate reduction or loss of DSBs and recombination frequency. Meiotic recombination is initiated by DSBs, whose formation requires multiple proteins. LinE proteins Rec10, Rec25, Rec27 and Mug20 form a putative LinE complex and are required for most DSBs at hotspots, to which they bind with high specificity^16, 19, 21^. Since the viable spore yields of the LinE mutants studied here were not strongly reduced (unpublished data), the observed recombination-deficiency of these mutants is likely a consequence of subdued complex formation or impaired DSB formation rather than inefficient DSB repair. Missense LinE mutants are a useful resource to address these aspects of DSB regulation and to identify protein domains involved in these processes.

The LinE mutants we identified reduced recombination to different levels: four *rec25* mutants – *rec25-234* (N36D, L137P), *rec25-235* (N63I), *rec25-236* (K124I), and *rec25-237* (L90P), one *rec27* mutant – *rec27-241* (Q119K), and one *mug20* mutant – *mug20-250* (L52P) had the null phenotype, indicating that these amino acids are critical for each protein’s function, although some of these amino acids are not conserved among *Schizosaccharomyces* species (Suppl. Fig. S4). As noted above, three *rec27* mutants – *rec27-238*, *rec27-239* and *rec27-240* – clustered in a small N-terminal region of Rec27 and reduced recombination frequencies moderately; similar reductions were observed in *mug20-251*.

Since the recombination-deficiencies in these LinE mutants spanned a range from null to nearly wild-type, we measured DSBs at the *ade6-3049* hotspot in these mutants. DSBs in mutants with strongly reduced recombination – *rec25-235* (N63I), *rec27-241* (Q119K) and *mug20-250* (L52P) – were reduced to background level. In other mutants with reduced but not abolished recombination – *rec27-238* (K28E), *rec27-240* (K36E) and *mug20-251* (V78E) – DSBs were correspondingly reduced but not abolished (Figs 4 and S7). The detectable DSBs in these mutants suggest that the putative Rec10-Rec25-Rec27-Mug20 complex formed and accumulated in the nucleus, but the complex’s binding-efficiency to hotspots may be inefficient and thereby reduce but not eliminate DSB formation. At the DSB hotspots *ade6-3049* and *mbs1*, DNA breaks are repaired 3- or 4-times more frequently with the sister chromatid than with the homolog^38–40^. We infer that in the *rec27-238*, *rec27-240* and *mug20-251* mutants DSBs are repaired with the same bias as in wild-type cells, since DSB and recombinant frequencies are reduced in parallel.

A KR cluster near the N-terminus of Rec27 is important for its function. Three *rec27* mutants changed K (lysine) or R (arginine) to E (glutamic acid): *rec27-238* (K28E), *rec27-239* (R32E), and *rec27-240* (K36E) are each separated by three amino acids and clustered near the N-terminus of Rec27. They partially reduced recombination (both gene conversion and crossing-over) (Fig. 2B; Suppl. Table S3) and partially reduced DSBs at *ade6-3049* (Figs 4 and S7). These three amino acids are identical in the Rec27 proteins of five *Schizosaccharomyces* species and may be part of a functional domain to perform the protein’s function.

Our data indicate complex interactions of the N- and C-terminal domains of Rec10. Rec25-GFP, Rec27-GFP and Mug20-GFP interdependently co-localize at about 15 distinct foci in meiotic nuclei; nuclear accumulation of the proteins and formation of foci depends on Rec10 ^11, 16^. In the absence of one or another LinE protein, there is no observed GFP accumulation in the nucleus, even though where tested the proteins’ abundance in the cells is similar to that in wild-type cells^11^. The nuclear foci of LinE proteins are unlikely to be single protein molecules visible in the microscope we used. Instead, these foci may represent multiple distant regions on a chromosome bound by LinE proteins and forming a cluster that locally regulates DSB formation (Fowler *et al*., unpublished data).

Rec10, but not the other LinE proteins, has a predicted nuclear localization signal (NLS) in the middle of the protein (K494-S503) (http://nls-mapper.iab.keio.ac.jp/cgi-bin/NLS_Mapper_form.cgi). In mitotic cells, ectopically expressed Rec10 strongly localizes in the nucleus, while Rec25 and Mug20 are distributed evenly over the cytoplasm and nucleus^41^. Rec25-Rec27-Mug20-Rec10 may form a complex and then be brought into the nucleus by Rec10. This view is consistent with Rec10, but not the other LinE proteins, having an obvious NLS. Alternatively, the different components may independently enter the nucleus and LinE complex formation may cause nuclear retention. Since Rec25, Rec27, and Mug20 are small (125–151 amino acids), they may freely diffuse into the nucleus but not accumulate there without association with Rec10.

The *rec10* mutations we used to investigate Rec25-GFP focus-formation did not occur in this predicted NLS. In these *rec10* mutants Rec25-GFP accumulated in the nucleus, although in the case of the N-terminal mutant *rec10-216* (R184F, D185T) less efficiently and with some delay (Fig. 5). This suggests that LinE complex formation or nuclear import is defective in this mutant, which affects two amino acids located in a region of Rec10 (H183-L191) identical in five *Schizosaccharomyces* species (Fig. S4D). The deficiency in localization of the LinE complex agrees with the recombination deficiency of the *rec10-216* mutant, which shows a strong reduction in recombination (gene conversion and crossing-over) nearly to the levels of *rec10Δ* (Table 2).

In another N-terminal mutant *rec10-109* (V176I, G178D), LinE formation assayed in chromosome spreads is considerably reduced^4^. In intact *rec10-109* cells, we observed that Rec25-GFP signal was concentrated in the nucleus with normal kinetics, suggesting that LinE complex formation and nuclear import or retention are normal but the signal did not evolve into dot-like foci as distinct as those in *rec10* ^+^ cells (Fig. 5). Gene conversion at the hotspot *ade6-M26* is reduced in *rec10-109* by a factor > 40, but, remarkably, crossing-over is not significantly reduced in two other intervals on another chromosome (Table 2)^23, 32, 34^. Genome-wide analysis showed that most of the DSBs at most hotspots, including *ade6-3049*, are strongly reduced but not eliminated in *rec10-109*; at some hotspots, however, DSBs are still prominent^16^. The defect in LinE focus formation agrees with the genome-wide analysis of DSBs in this mutant. The LinE complex’s binding to hotspots may be defective in the *rec10-109* mutant, and the recombination still observed in *rec10-109* mutants may originate mostly from DSBs outside of hotspots^16^.

A different Rec25-GFP signal was observed in intact cells of the C-terminal *rec10-144* (G727E) mutant. A few distinct foci formed in the nucleus although later than, and not as efficiently as, in wild-type cells. The Rec25-GFP nuclear foci in *rec10-144* may be limited to a set of hotspot clusters noted above; alternatively, binding of the LinE complex to hotspots may be unstable, hampering normal levels of DSB formation at the hotspot.

In the *rec10-109* (V176I, G178D) mutant, the residual gene conversion at the hotspot *ade6-M26* and crossing-over in the intervals tested (*lys3 – ura1* and *ura1 – met5*) was Rec25-dependent: deletion of *rec25* in *rec10-109* reduced gene conversion to the *rec10Δ* level and crossing over to the *rec25Δ* level (Table 2). This result indicates that there is still some LinE-dependent recombination activity in *rec10-109*. Perhaps LinE complexes still load onto some hotspots but at such a low level that they cannot be as readily detected by Rec25-GFP fluorescence as in *rec10* ^+^ cells; alternatively, hotspot clustering may be defective (see above). Unlike the N-terminal mutant *rec10-109*, the C-terminal mutant *rec10-155* (E691I and frame-shift) did not manifest this genetic interaction with *rec25Δ*: the recombinant frequencies in the double mutant *rec10-155 rec25Δ* were the same as that in each single mutant, which are similar (Table 2). This suggests that Rec25 interacts with the Rec10 C-terminus, and that this interaction is lost in the truncated Rec10-155 protein. The similar recombination phenotypes of the C-terminal *rec10-144* (G727E) and *rec25Δ* mutants and the mild effect of *rec25Δ* in *rec10-144* background are also consistent with Rec25 interacting with this region of Rec10.

Our study shows that *rec10* mutations altering either the N-terminal region (*rec10-109*; V176I, G178D) or the C-terminal region (*rec10-144*; G727E) are defective in LinE focus formation. This result and the intragenic complementation of N-terminal and C-terminal missense *rec10* mutations (Table 2)^22, 32^ suggest that Rec10 protein is self-interacting. There may be domains provided from different Rec10 molecules in a dimeric or higher order complex. In addition, the C-terminal region appears to interact with Rec25 for DSB formation. Two adjacent residues (R184, D185) in the N-terminal region may have a role in LinE complex formation or nuclear import. In summary, the *rec10* mutants studied here differ from *rec10* ^+^ with respect to their interactions with Rec25, assayed both genetically and microscopically, but no clear pattern has yet emerged to explain these alterations.

The eleven novel missense mutations of LinE proteins reported here will help shed more light on LinE complex organization, regulation, and functions of the SC in DSB formation to form meiotic crossovers.

## Materials and Methods

### *S. pombe* strains, growth media, and genetic methods

Suppl. Table S1 lists the *S. pombe* strains used in this study. Media for cell growth and methods for meiotic crosses and random spore analysis were described^42^.

### *S. pombe* plasmids, oligonucleotides, and site-directed mutant construction

*rec25-256::ura4* ^+^, *rec27-257::ura4* ^+^ and *mug20-258::ura4* ^+^ were constructed using the method described^43^. Briefly, the *ura4* ^+^ ORF (open reading frame) was amplified from plasmid pFY20^30^ using primers OL3266 and OL3267 (Suppl. Table S2) with 80 bp of homology to DNA flanking the *rec25* ORF, or OL3268 and OL3269 with 80 bp of homology to DNA flanking the *mug20* ORF. For *rec27-257::ura4* ^+^construction, a 1.8 kb DNA fragment containing *ura4* ^+^^44^ with ~250 bp of homology to DNA flanking each side of the *rec27* ORF, as formerly annotated, was generated in two steps by DNA polymerase chain-reactions (PCRs): first, ~250 bp DNA fragments upstream or downstream of the former *rec27* ORF were amplified in PCRs using OL3441 with OL3438, or OL3439 with OL3440, respectively, and wild-type (strain GP13) genomic DNA. Then, the purified PCR products together with pFY20 as template were used to amplify the 1.8 kb *ura4* ^+^ by a secondary PCR using primers OL3442 and OL3443. Since the PCR products from the first step overlap with the 5′ and 3′ ends of *ura4* ^+^ on plasmid pFY20, the second step PCR generated *ura4* ^+^ with ~250 bp of homology to regions flanking *rec27*. The PCR products containing *ura4* ^+^ and homology to regions flanking *rec25*, *rec27* and *mug20* were used to transform strain GP4915 (*ura4-D18*) to uracil prototrophy. Integration at the *rec25* locus was verified by a PCR using primers OL326 and OL3258; OL326 and OL3260 for *mug20*; and OL3255 and OL3256 for *rec27*.

Plasmids pLM06, pLM08 and pLM09 with wild-type *rec25* ^+^ *, rec27* ^+^ and *mug20* ^+^, respectively, were constructed as follows. The *rec25* ^+^ gene with 297 bp of 5′ and 1118 bp of 3′ flanking DNA was amplified from wild-type (strain GP13) genomic DNA using OL3250 and OL3251 as primers, and the PCR fragment was cloned into pFY20 at the *Xma*I site to produce plasmid pLM06. The *rec27* ^+^ gene with 292 bp of 5′ and 314 bp of 3′ flanking the *rec27* ORF, as formerly annotated, was PCR-amplified using OL3248 and OL3249 as primers and cloned into pFY20 at the *Kpn*I site to produce pLM08. The *mug20* ^+^ gene with 291 bp of 5′ and 597 bp of 3′ flanking DNA was PCR-amplified using OL3253 and OL3254 as primers and cloned into pFY20 at the *Kas*I site to produce pLM09.

The mutations *rec27-239*, *rec27-242*, *rec27-243*, *rec27-244*, and *rec27-245* were introduced into plasmid pLM08 by mutagenesis using the QuikChange Lightning Site-Directed Mutagenesis Kit (Agilent catalog # 210518). Primers used to introduce point mutations were: OL3399 and OL3400 for *rec27-239*; OL3401 and OL3402 for *rec27-242*; OL3403 and OL3404 for *rec27-243*; OL3405 and OL3406 for *rec27-244*; and OL3407 and OL3408 for *rec27-245*. After sequencing to confirm the mutations, the mutated *rec27* ORFs with flanking homologous sequences were released from the plasmids by *Kpn*I digestion and integrated into the chromosome at the *rec27* locus by transformation of strain GP8513 (*rec27-257::ura4* ^+^) to 5-fluorouracil (FOA)-resistance. Integration at the *rec27* locus was identified by a PCR using primers OL3255 and OL3256.

*rec10-216* was generated as follows: primers OL2502 and OL2503, each of which contains six random nucleotides in place of the wild-type codons for R184 and D185, were used to generate randomly mutagenized R184, D185 mutations in *rec10*. Plasmid pYL176^34^, bearing the wild-type *rec10* gene with ~550 bp of 5′ and ~270 bp of 3′ flanking DNA, was used as a template. After PCR using the QuikChange Lightning Site-Directed Mutagenesis Kit (Agilent catalog # 210518) and *Dpn*I digestion to remove the template plasmid DNA, the mutant plasmids were introduced into ultracompetent *Escherichia coli* XL10-Gold cells {∆(*mcrA*)*183 ∆*(*mcrCB-hsdSMR-mrr*)*173 endA1 supE44 thi-1 recA1 gyrA96 relA1 lac* Hte [F′ *proAB lacI* ^*q*^ *Z∆M15 Tn10* (Tet^R^) Amy Cam^R^)]} (Agilent) by transformation to ampicillin-resistance. Plasmids were isolated from seven isolated colonies and sequenced using OL1192; three were mutant: R184V, D185*; R184F, D185T; and R184I, D185S. (*Indicates a non-sense mutation.) Strain GP6993 (*rec10-175::kanMX6*) was transformed with the mutated plasmids and crossed with GP6994 (*rec10-175::kanMX6*) to assay intergenic and intragenic recombination. The missense mutation R184F, D185T (*rec10-216*) showed strong recombination deficiency, equal to that of the nonsense mutation R184V, D185* (*rec10-261*) (data not shown). The *rec10-216* ORF with flanking homologous sequences was released from the plasmid by *Pvu*II and *Nsi*I digestion and integrated into the chromosome at the *rec10* locus by transformation of strain GP7301 (*rec10-260::ura4* ^+^) to 5-fluorouracil (FOA)-resistance. Integration at the *rec10* locus was verified by a PCR using primers OL1778 and OL1779. GP7301 (*rec10-260::ura4* ^+^) was constructed by transformation of strain GP6994 to Ura^+^ using a 1.8 kb *ura4* ^+^ PCR fragment with 80 bp of homology to DNA flanking the *rec10* ORF [amplified from pFY20 which contains *ura4* ^+^^30^ using OL2722 and OL2723 as primers]. Integration at the *rec10* locus was verified by PCR using primers OL1778 and OL1779.

### *rec25, rec27* and *mug20* mutant library construction

Random mutations were introduced into each LinE ORF using the Genemorph II EZclone Domain Mutagenesis Kit (Agilent catalog # 200552). Oligos OL3257 and OL3258 for *rec25*, OL3255 and OL3256 for *rec27*, and OL3259 and OL3260 for *mug20* were used to synthesize gene fragments containing random mutations using pLM06, pLM08 or pLM09, respectively, as templates. After denaturation and annealing to pLM06, pLM08 or pLM09, the mutated PCR products served as “mega-primers” for a PCR with a specialized high-fidelity enzyme mix in the kit. After *Dpn*I digestion to remove the template plasmid DNA, the mutant plasmids were introduced into ultracompetent *E. coli* XL10-Gold cells by transformation to ampicillin-resistance. DNA from separate pools of ~6500 transformants for *rec25*, ~4000 transformants for *rec27*, and ~2800 transformants for *mug20* generated the *rec25*, *rec27* and *mug20* mutant libraries. This DNA was used to transform diploid strains GP8370 (*rec25Δ*), GP8367 (*rec27Δ*), and GP8484 (*mug20Δ*) to uracil prototrophy by selection on EMM2 + Ade plates incubated at 32 °C for 5–6 days to allow growth into visible colonies, mating, and sporulation. Single colonies were picked into 75 μl of sterile water in a sterile 96-well plate, and 4 μl of the cell suspension was spotted onto NBA + Ade “keeper” plates. Then, 25 μl of glusulase (1:50 dilution in water) was added to each well. After overnight incubation at 32 °C 100 μl of 60% ethanol was added to each well and held at room temperature for 30 min. The plates were centrifuged at 4000 rpm (2880× g) for 4 min in an Eppendorf centrifuge model 5810; the cells were washed twice with 125 μl of sterile water and suspended in 125 μl of sterile water. Aliquots of 4 μl of the spore suspensions were spotted on YEA plates and incubated for 4 days at 32 °C to assay the Ade^+^ recombinant frequencies (Fig. 1).

The *rec27* mutant genes were put into the chromosome as for the site-directed *rec27* mutant genes described above. The *rec25* mutant genes with flanking DNA were released by *Kpn*I and *Xma*I digestion and used to transform GP8210 (*rec25-256::ura4* ^+^) to FOA-resistance; a PCR using OL3257 and OL3258 as primers identified homologous replacements. The *mug20* mutant genes were released by *Kas*I digestion and similarly transferred to strain GP8211 (*mug20-258::ura4* ^+^) and identified by a PCR using OL3259 and OL3260 as primers.

### Assay for meiotic DSBs

Meiotic induction and DNA analysis were described by^45, 46^. DNA in agarose plugs was digested with *Bsr*G1 overnight at 37 °C, subjected to electrophoresis through 0.8% agarose, and transferred to a nylon membrane (Zeta-probe GT membrane, Biorad). DNA broken at the *ade6-3049* DSB hotspot was detected by hybridization to a 1 kb probe from the right end of the 10.1 kb *Bsr*GI fragment. The probe was a PCR product amplified from wild-type genomic DNA using OL3693 and OL3694 as primers and labeled with [α-^32^P] dCTP (3000 Ci/mmol). Signals were detected using a Typhoon storage PhosphorImaging system (GE Healthcare).

### Fluorescence microscopy

Diploid *pat1-114 rec10* mutant cells producing Rec25 with GFP fused to its C-terminus (*rec25-204::GFP*) were induced for meiosis, and the GFP signals were examined as described^16^. Cells were fixed sequentially with 100% methanol and 100% acetone before and at different times after induction of meiosis. The cell suspension was spread on a poly-L-lysine coated slide, acetone allowed to evaporate, and mounting solution (Vectashield; Vector Laboratories, Inc.) supplemented with DAPI (2 μg/ml) added. Images are maximal projections of 9–11 sections, step size of 0.4 mm, to cover the whole cell (approx. 4 µm total). DNA images are a single focal plane, because out-of-focus DAPI fluorescence obscures the projection. Images were obtained with a Nikon Eclipse 90i microscope equipped with a 100/1.4 Oil Plan APO VC lens, a Hamamatsu ORCAER camera, and MetaMorph software (Molecular Devices).

## Electronic supplementary material

Supplementary information
