# Supplementary material for: Functional organization of protein determinants of meiotic DNA break hotspots
Source: Sci Rep. 2017 May 3;7:1393. doi: 10.1038/s41598-017-00742-3 (PMC5431104; doi:10.1038/s41598-017-00742-3)
Supplement: Supplementary file 1 — Supplementary information [file 41598_2017_742_MOESM1_ESM.pdf]

Supplementary Figures and Tables for

Functional organization of protein determinants of meiotic DNA break hotspots

Lijuan Ma[1], Kyle R. Fowler[1,2] , Cristina Martín-Castellanos[3] and Gerald R. Smith[1,*]

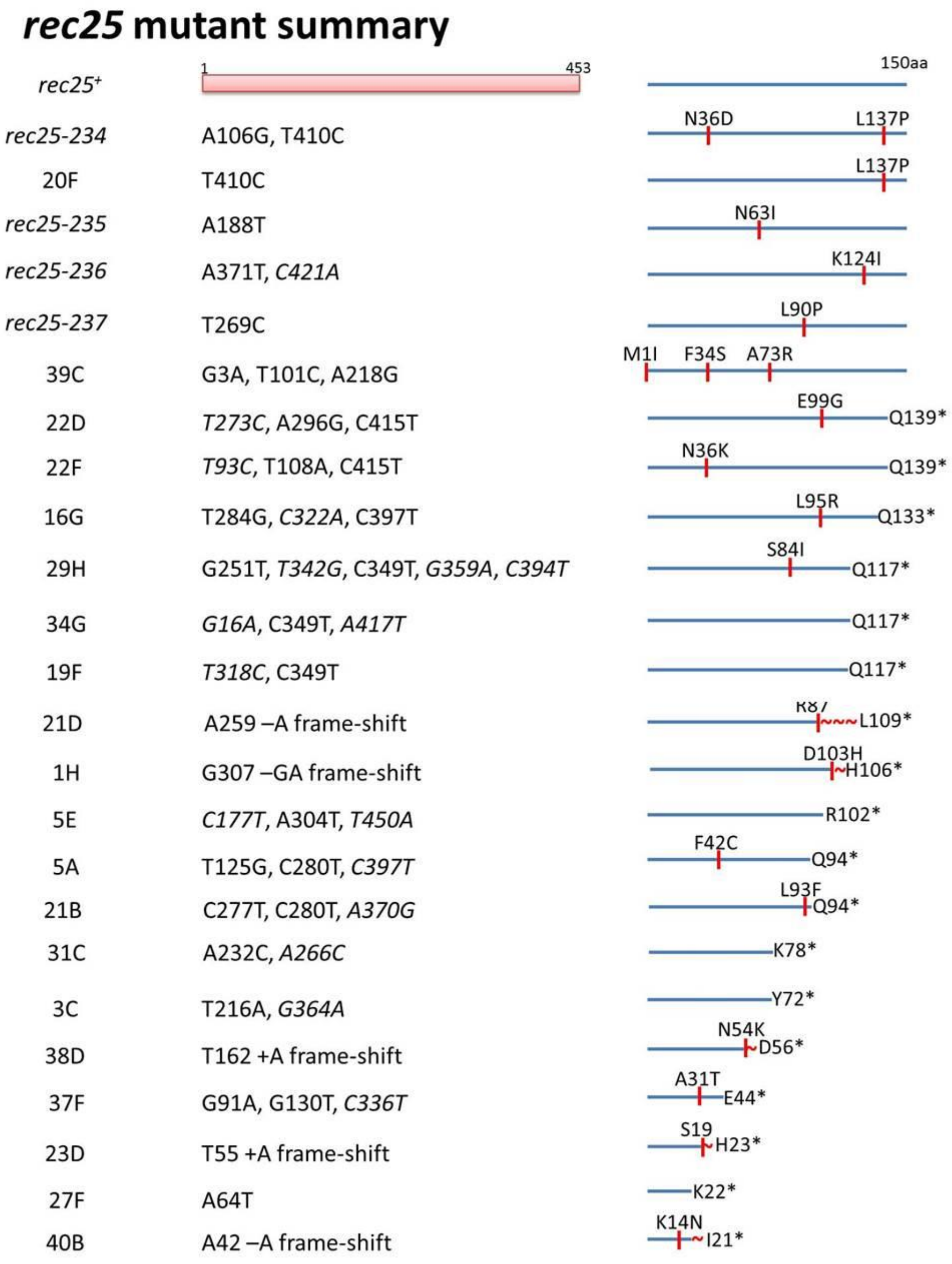

Supplementary Figure S1. Summary of *rec25* mutants. Mutants listed here were from the screen and were recombination-negative in qualitative tests (low frequency of white papillae on red spots (Figure 1). Nucleotide changes in italics do not change protein coding. * Non-sense mutation, ~ frame-shift. The mutants without an assigned allele number were not tested further. See main text for analysis of other mutants. Pink box is the exon.

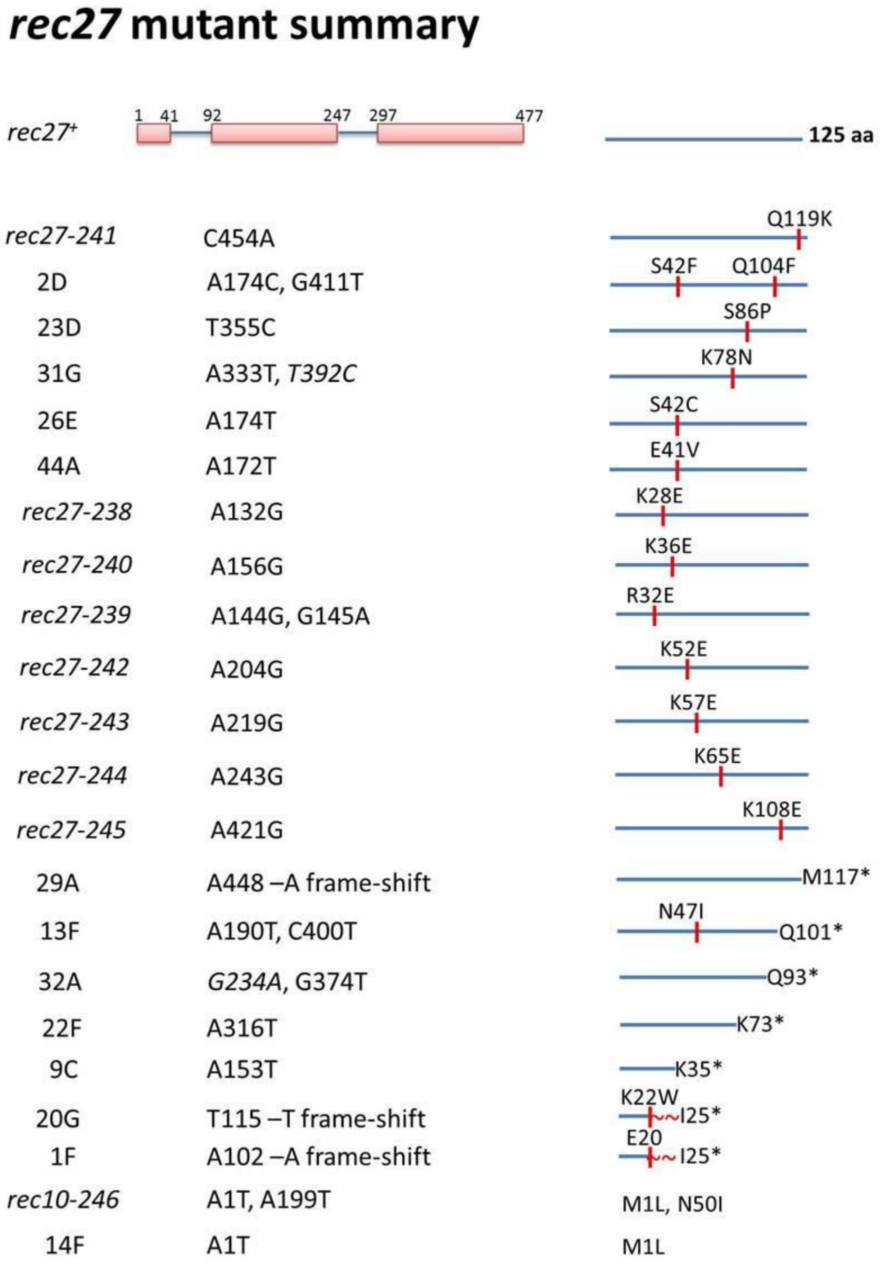

Supplementary Figure S2. Summary of *rec27* mutants. Mutants listed here were from the screen (except for *rec27-239*, *242*, *243*, *244* and *245* which were from site-directed mutagenesis) and were recombination-negative in qualitative tests (low frequency of white papillae on red spots (Figure 1). Nucleotide changes in italics do not change protein coding. * Non-sense mutation, ~ frame-shift. The mutants without an assigned allele number were not tested further. See main text for analysis of other mutants. Pink boxes are exons.

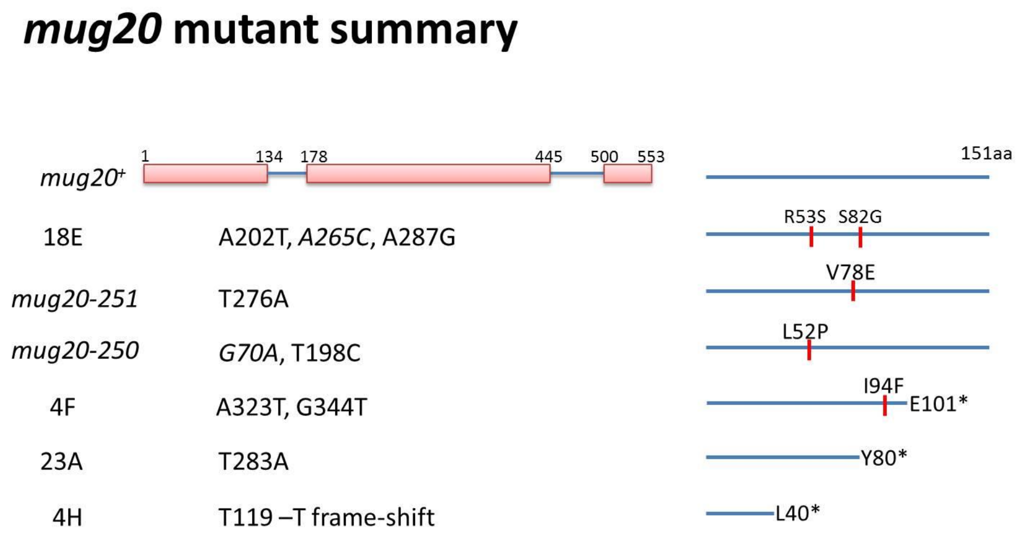

Supplementary Figure S3. Summary of *mug20* mutants. Mutants listed here were from the screen and were recombination-negative in qualitative tests (low frequency of white papillae on red spots (Figure 1). Nucleotide changes in italics do not change protein coding. * Non-sense mutation. The mutants without an assigned allele number were not tested further. See main text for analysis of other mutants. Pink boxes are exons.

A

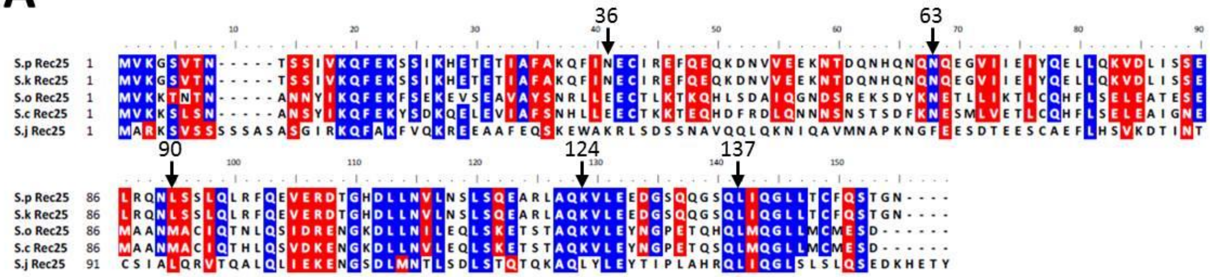
36
63
90
124
137
S.p Rec25
S.k Rec25
S.o Rec25
S.c Rec25
S.j Rec25

B

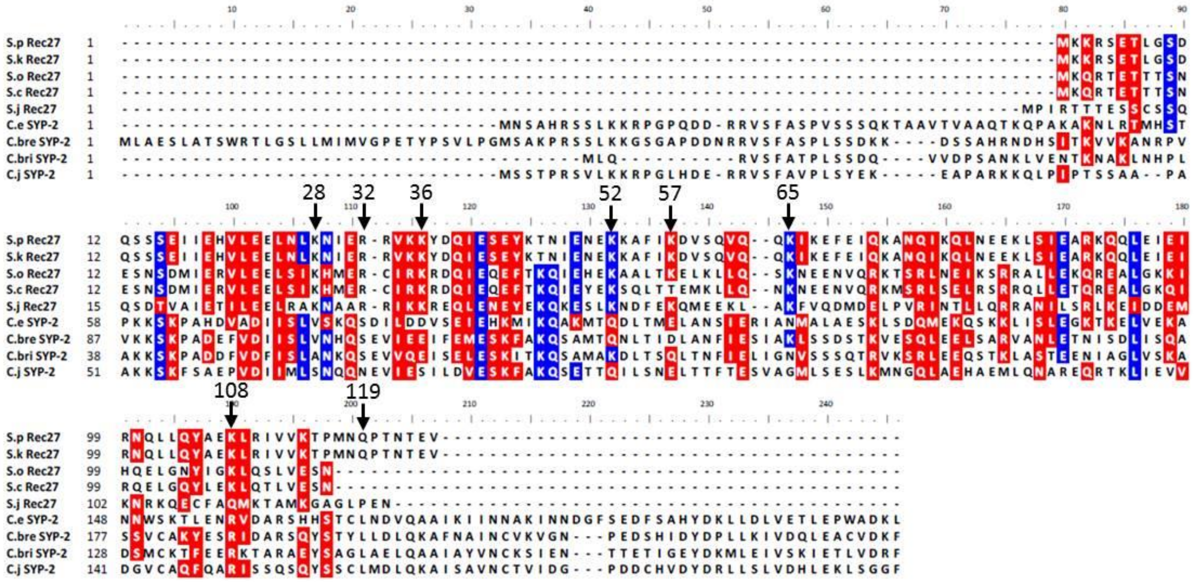
28
32
36
52
57
65
108
119
S.p Rec27
S.k Rec27
S.o Rec27
S.c Rec27
S.j Rec27
C.e SYP-2
C.bre SYP-2
C.bri SYP-2
C.j SYP-2

C

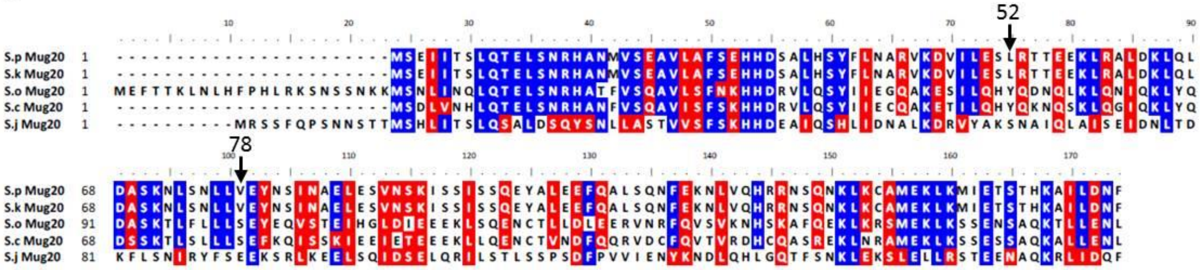
52
78
S.p Mug20
S.k Mug20
S.o Mug20
S.c Mug20
S.j Mug20

D

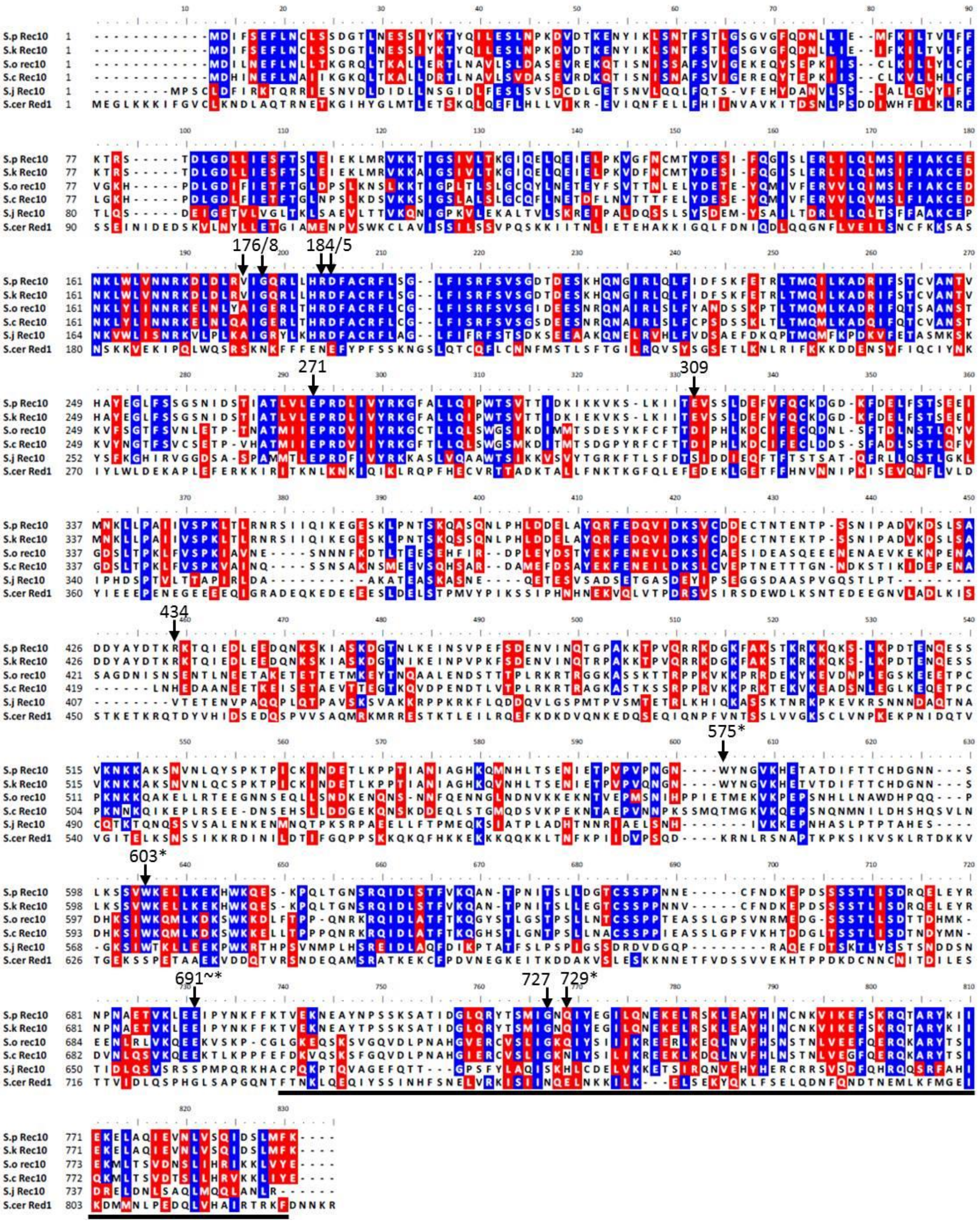

Supplementary Figure S4. Similarity between amino acid sequences of *S. pombe* Rec25, Rec27, Mug20 and Rec10 and proteins from other *Schizosaccharomyces* spp., *Caenorhabditis* spp. and *Saccharomyces cerevisiae.* The amino acid sequence alignment was performed in BioEdit using ClustalW [1]. Amino acids with similar physicochemical properties

are shaded in red (BLOSUM62 similarity matrix with 66% threshold). Identical amino acids are shaded with blue. Gaps are indicated with hyphens. Positions (numbered as in *S. pombe*) of amino acids changed in the mutants studied here are indicated by vertical arrows, * Non-sense mutation, ~ frame-shift. *Schizosaccharomyces* spp. are abbreviated as follows: S.p, *S. pombe*; S.k, *S. kambucha*; S.c, *S. cryophilus*; S.o, *S. octosporus*; S.j, *S. japonicus*. *Schizosaccharomyces* species are shown in their order of evolutionary distance [2]. *Saccharomyces cerevisiae* is abbreviated as *S.cer*. *Caenorhabditis* spp. are abbreviated as follows: C.e, *C. elegans*; C.bre, *C. brenneri*; C.bri, *C. briggsae*; C.j, *C. japonica*. The region of *Saccharomyces cerevisiae* Red1 with amino acid similarity to Rec10 is underlined in panel D.

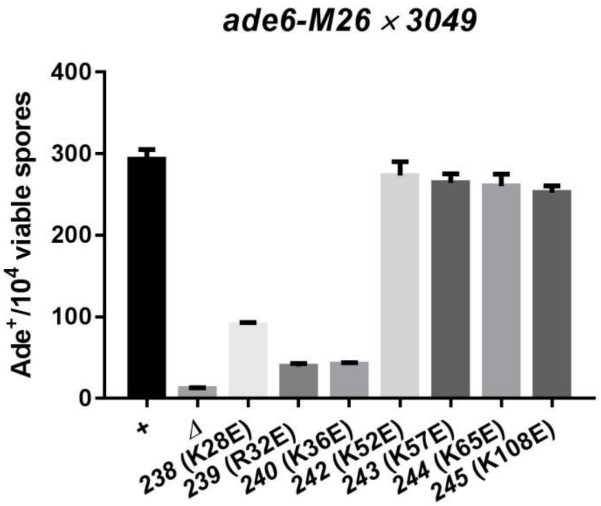

Supplementary Figure S5. Clustered *rec27* mutants with conserved positively charged arginine or lysine, changed to negatively charged glutamate, are recombination-deficient. Meiotic recombination between *ade6-M26* and *ade6-3049* was assayed in the indicated mutants. The indicated amino acids are conserved in *Schizosaccharomyces* spp. (*rec27-239* and *rec27-244*) and in *Caenorhabditis* spp. (*rec27-242*, *rec27-243* and *rec27-245*) (Figure S4B). Data (mean ± SEM; n=4 except for $rec27^{+}$ and *rec27Δ*, for which n=12) are from Suppl. Table S3; *rec27-238* and *rec27-240* data from Figure 2B are shown for comparison.

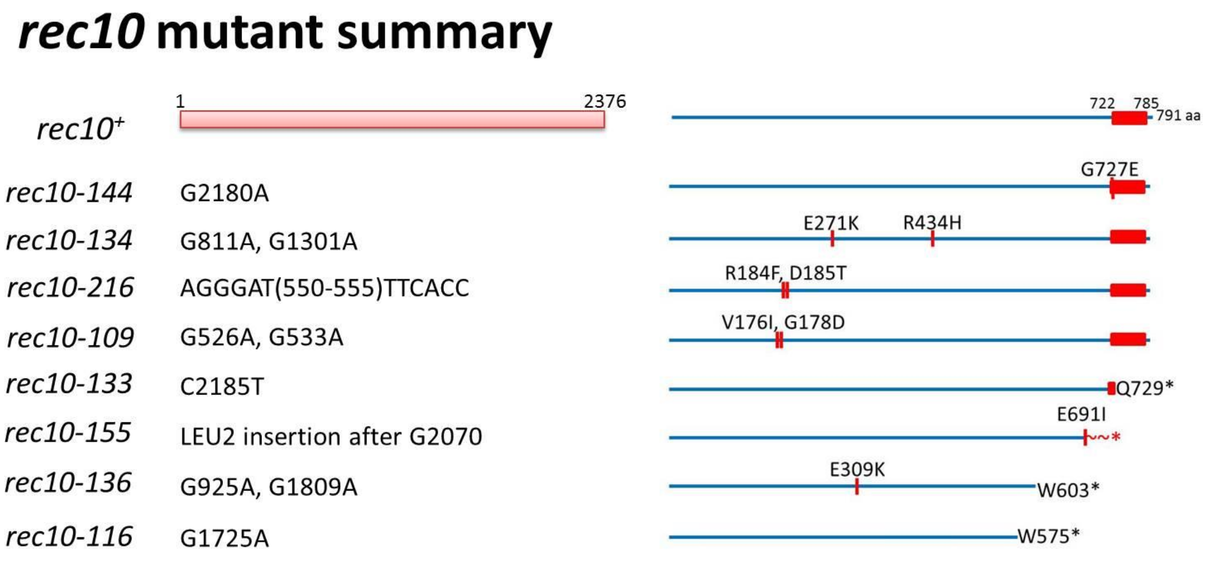

Supplementary Figure S6. Summary of *rec10* mutants. *rec10-216* was Isolated by random mutagenesis of two codons (R184 and D185). Other mutants were previously described [3-5]. * Non-sense mutation, ~ frame-shift. Red box indicates the region (722 – 785 aa) of similarity with *S. cerevisiae* Red1 [6]. Pink box is the exon.

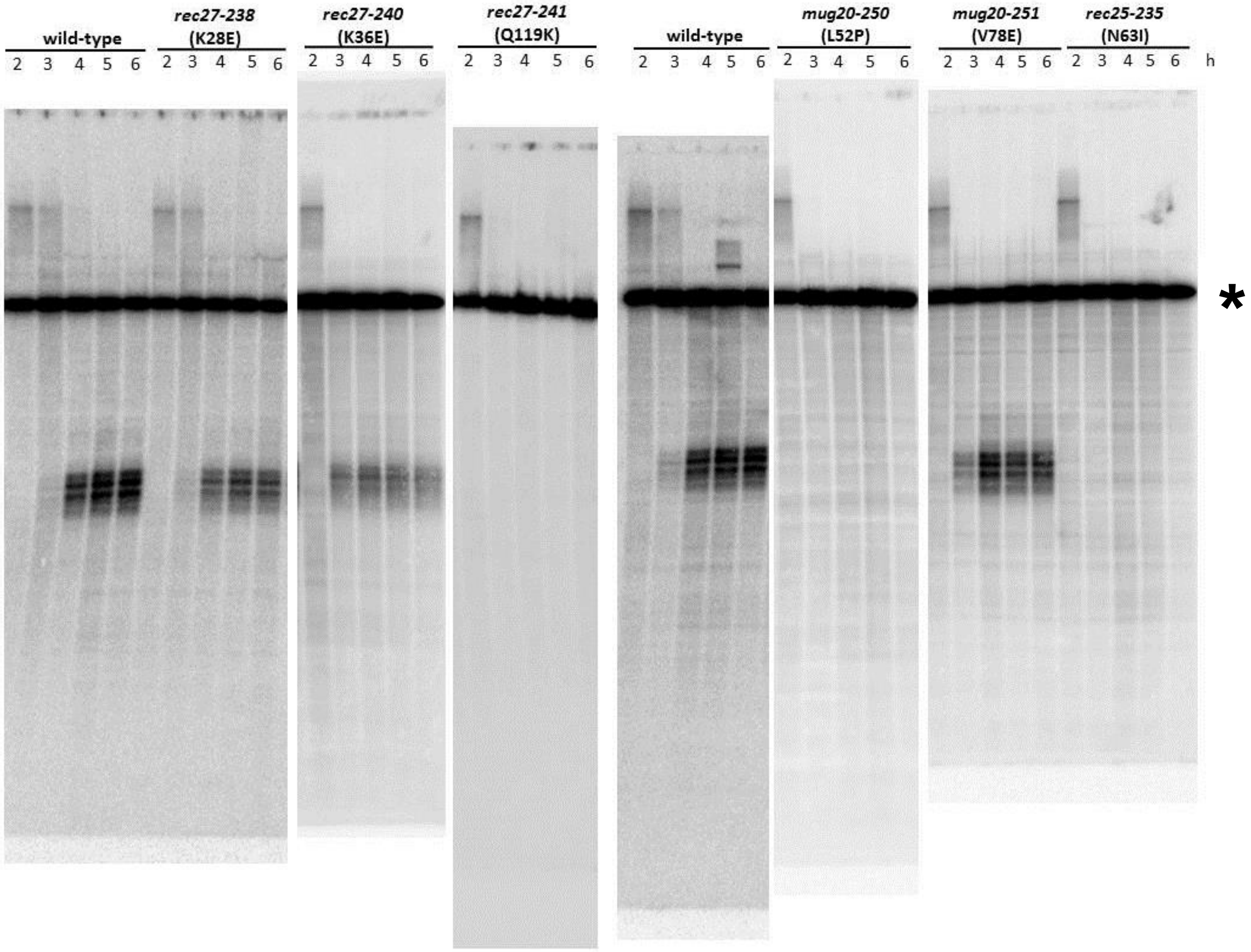

Supplementary Figure S7. Meiotic DSBs of LinE mutants. Shown are full-length blots of the gels corresponding to the data in Figure 4. Note that at 2 and 3 h, replication intermediates (Y-shaped molecules, etc.) migrate more slowly than the linear 10.1 kb restriction fragment (*), whose position was determined relative to ethidium bromide-stained markers (not visible in these autoradioagraphs). In the wild-type 5 h lane on the right, low-level partial digestion products also migrate more slowly. The irregular bands near the tops of the gels are the wells into which the DNA was loaded.

Supplementary Table S1. *S. pombe* strains

Strain	Genotype	Used in
GP13	h^- *ade6-52*	pLM06, pLM08 and pLM09 construction
GP20	h^+ *leu1-32*	Wild-type *rec27*$^+$ sequencing
GP1023	h^- *ade6-52 rec10-109 ura1-171*	Table 2
GP4414	h^+ *ade6-M26 arg1-14 rec10-175::kanMX6*	Table 2
GP4915	h^- *ade6-M26 ura4-D18 arg1-14*	Construction of *rec25-256::ura4*$^+$, *rec27-257::ura4*$^+$ and *mug20-258::ura4*$^+$
GP5366	h^+ *ade6-3049 pat1-114 rad50S*	Figures 4 and S7
GP5570	h^+ *ade6-M26 lys3-37 met5-1*	Table 2
GP6294	h^- *ade6-52 ura1-171 rec25-180::kanMX6*	Table 2
GP6295	h^+ *ade6-52 ura1-171 rec25-180::kanMX6*	Figure 3A, Table 1 and Table S3
GP6297	h^- *ade6-52 ura1-171*	Table 2
GP6323	h^+ *ade6-M26 lys3-37 met5-1 rec25-180::kanMX6*	Table 2
GP6558	h^+ *ade6-M26 lys3-37 met5-1 rec10-155*	Table 2
GP6912	h^+ *ade6-M26 lys3-37 met5-1 rec10-109*	Table 2
GP6913	h^- *ade6-52 ura1-171 rec10-109*	Table 2
GP6914	h^+ *ade6-M26 lys3-37 met5-1 rec10-116*	Table 2
GP6915	h^- *ade6-52 ura1-171 rec10-116*	Table 2
GP6916	h^- *ade6-52 ura1-171 rec10-155*	Table 2
GP6917	h^+ *ade6-M26 lys3-37 met5-1 rec10-175::kanMX6*	Table 2
GP6918	h^- *ade6-52 ura1-171 rec10-175::kanMX6*	Table 2
GP6963	h^+ *ade6-M26 lys3-37 met5-1 rec10-144*	Table 2
GP6964	h^- *ade6-52 ura1-171 rec10-144*	Table 2
GP6993	h^+ *ade6-M26 rec10-175::kanMX6 ura4-D18 lys3-37 met5-1*	Construction of *rec10-216*
GP6994	h^- *ade6-52 ura4-D18 rec10-175::kanMX6*	Construction of *rec10-216 and rec10-260::ura4*$^+$
GP6996	h^+ *ade6-M26 lys3-37 met5-1 rec10-134*	Table 2
GP6997	h^- *ade6-52 ura1-171 rec10-134*	Table 2
GP7301	h^- *ade6-52 ura4-D18 rec10-260::ura4*$^+$	Construction of *rec10-216*
GP7491	h^+ *ade6-52 mug20::natMX6*	Figure 3C, Table 1 and Table S3
GP7714	h^- *ade6-52 ura4-D18 rec10-216*	Table 2
GP7747	h^- *ade6-M26 arg1-14*	Figures 2, 3 and S6, Table 1 and Table S3
GP8210	h^- *ade6-M26 ura4-D18 arg1-14 rec25-256::ura4*$^+$	Figure 3A, Table 1 and Table S3
GP8211	h^- *ade6-M26 ura4-D18 arg1-14 mug20-258::ura4*$^+$	Figure 3C, Table 1 and Table S3
GP8325	h^+ *ade6-3049 rec25-180::kanMX6*	Figure 3A, Table 1 and Table S3
GP8365	h^+ *ade6-52 ura4-D18 rec27::KanMX6 lys4-95*	Figure 3B, Table 1 and Table S3
GP8367	h^+/h^- *ade6-3049/ade6-M26 ura4-D18/ura4-D18 rec27::kanMX6/rec27::kanMX6 lys4-95/+ +/his4-239*	Figure 1
GP8370	h^+/h^- *ade6-3049/ade6-M26 ura4-D18/ura4-D18 rec25::kanMX6/rec25::kanMX6 lys4-95/+ +/his4-239*	Figure 1

GP8484	h^+/h^- *ade6-3049/ade6-M26 ura4-D18/ura4-D18 mug20::natMX6/mug20::natMX6lys4-95/+ +/his4-239*	Figure 1
GP8513	h^- *ade6-M26 ura4-D18 arg1-14 rec27-257::ura4*$^+$	Figures 2A, 3B, Table 1 and Table S3
GP8527	h^- *ade6-M26 ura4-D18 arg1-14 rec27-247*	Figure 2A, Table 1 and Table S3
GP8528	h^- *ade6-M26 ura4-D18 arg1-14 rec27-248*	Figure 2A, Table 1 and Table S3
GP8530	h^- *ade6-M26 ura4-D18 arg1-14 rec27-246*	Figure 2A, Table 1 and Table S3
GP8532	h^- *ade6-M26 ura4-D18 arg1-14 rec27-238*	Figure 3B, Table 1 and Table S3
GP8533	h^- *ade6-M26 ura4-D18 arg1-14 rec27-249*	Figure 2A, Table 1 and Table S3
GP8534	h^- *ade6-M26 ura4-D18 arg1-14 rec27-240*	Figure 3B, Table 1 and Table S3
GP8555	h^+ *ade6-3049 ura4-D18 rec27::kanMX6 lys4-95*	Figures 2A, 3B and S6, Table 1 and Table S3
GP8587	h^- *ade6-M26 ura4-D18 arg1-14 rec27-243*	Figure S1, Table 1 and Table S3
GP8588	h^- *ade6-M26 ura4-D18 arg1-14 rec27-244*	Figure S1, Table 1 and Table S3
GP8589	h^- *ade6-M26 ura4-D18 arg1-14 rec27-245*	Figure S1, Table 1 and Table S3
GP8623	h^- *ade6-M26 ura4-D18 arg1-14 rec27-241*	Figure 3B, Table 1 and Table S3
GP8635	h^- *ade6-M26 ura4-D18 arg1-14 rec27-239*	Figure S1, Table 1 and Table S3
GP8636	h^- *ade6-M26 ura4-D18 arg1-14 rec27-242*	Figure S1, Table 1 and Table S3
GP8640	h^- *ade6-M26 ura4-D18 arg1-14 rec25-234*	Figure 3A, Table 1 and Table S3
GP8641	h^- *ade6-M26 ura4-D18 arg1-14 rec25-235*	Figure 3A, Table 1 and Table S3
GP8642	h^- *ade6-M26 ura4-D18 arg1-14 rec25-236*	Figure 3A, Table 1 and Table S3
GP8644	h^- *ade6-M26 ura4-D18 arg1-14 rec25-237*	Figure 3A, Table 1 and Table S3
GP8658	h^- *ade6-M26 ura4-D18 arg1-14 mug20-251*	Figure 3C, Table 1 and Table S3
GP8659	h^- *ade6-M26 ura4-D18 arg1-14 mug20-250*	Figure 3C, Table 1 and Table S3
GP8689	h^+ *ade6-3049 mug20::natMX6*	Figure 3C, Table 1 and Table S3
GP8914	h^+ *ade6-3049 pat1-114 rad50S rec27-241*	Figures 4 and S7
GP8970	h^+ *ade6-3049 pat1-114 rad50S rec27-238*	Figures 4 and S7
GP8971	h^+ *ade6-3049 pat1-114 rad50S rec27-239*	Figures 4 and S7
GP9024	h^+ *ade6-3049 pat1-114 rad50S mug20-250*	Figures 4 and S7
GP9025	h^+ *ade6-3049 pat1-114 rad50S mug20-251*	Figures 4 and S7
GP9026	h^+ *ade6-3049 pat1-114 rad50S rec25-235*	Figures 4 and S7
GP9295	h^+ *ade6-M26 rec25-180::kanMX6 rec10-144 lys3-37*	Table 2
GP9253	h^- *ade6-52 rec25-180::kanMX6 rec10-144 ura1-171*	Table 2
KF1	h^+ *ade6-M26 lys3-37 met5-1 rec10-109 rec25-180::kanMX6*	Table 2
KF2	h^- *ade6-52 ura1-171 rec10-109 rec25-180::kanMX6*	Table 2
CMC78	h^-/h^- *pat1-114/pat1-114 ade6-M210/ade6-M216 leu1-32/leu1-32 rec25-204::GFP-kanMX6/rec25-204::GFP-kanMX6*	Figure 5
CMC841	h^-/h^- *pat1-114/pat1-114 ade6-52/ade6-M210 leu1-32/leu1-32 rec25-204::GFP-kanMX6/rec25-204::GFP-kanMX6 rec10-109/rec10-109*	Figure 5
CMC844	h^-/h^- *pat1-114/pat1-114 ade6-52/ade6-M210 leu1-32/leu1-32 rec25-204::GFP-kanMX6/rec25-204::GFP-kanMX6 rec10-144/rec10-144*	Figure 5
CMC851	h^-/h^- *pat1-114/pat1-114 ade6-52/ade6-M210 leu1-*	Figure 5

	32/leu1-32 rec25-204::GFP-kanMX6/rec25-204::GFP-kanMX6 rec10-216/rec10-216	

Alleles other than commonly used auxotrophies and mating type are described in the following references: *ade6-3049* [7], *ade6-M26* [8], *mug20::natMX6* [9], *pat1-114* [10], *rad50S* [11], *rec10-109* [4], *rec10-116* [3], *rec10-133* [3], *rec10-134* [3], *rec10-136* [3], *rec10-144* [12], *rec10-175::kanMX6* [13], *rec25-180::kanMX6* [14], *rec25-204::GFP-kanMX6* [5], and *rec27-184::kanMX6* [14]. *mug20-258::ura4*$^{+}$, *rec10-216*, *rec10-260::ura4*$^{+}$, *rec25-256::ura4*$^{+}$, *rec27-257::ura4*$^{+}$, and other *rec25*, *rec27* and *mug20* alleles were isolated in this study.

Supplementary Table S2. Oligonucleotides

Name	Sequence (5'-3')	Used for
OL326	AATTGTTGGCTTTGATGGAAG	*rec25-256::ura4*$^{+}$and *mug20-258::ura4*$^{+}$identification
OL1192	GCAAGAAATTGAACTGCCGAAAGTCGG	*rec10-216* sequencing
OL1778	GTCAAGCTCGAGGAAATACC	*rec10-216* and *rec10-260::ura4*$^{+}$identification
OL1779	TACCCCTGTCTTCTGCATTG	*rec10-216* and *rec10-260::ura4*$^{+}$identification
OL2502	CGAGTAATTGGTCAACGCTTGCTTCACNNNNNNNTTCG CATGTCGCTTCTTAAGCGGTCTA	*rec10-216* construction
OL2503	TAGACCGCTTAAGAAGCGACATGCGAANNNNNNNGTG AAGCAAGCGTTGACCAATTACTCG	*rec10-216* construction
OL2722	TAACGCTTCCAATGTGAATTGTTTTAAAGTAATATGTC AATATAAGCCAATATCAATAAATTTGATACTATAGGTT CAAGAGCTTGTGATATTGACGAAA	*rec10-260::ura4*$^{+}$ construction
OL2723	ATCAACTGAAACCGTTTTACGTATTTAATCCTATTTATT ATTCCAAAAAAAATCTATTAACACTTAAACGTAACTAA AATAGCTTAGCTACAAATCCCAC	*rec10-260::ura4*$^{+}$ construction
OL3248	GACTGAGGTACCAGAAGGCCACAAATGGTAAGA	pLM08 construction
OL3249	GACTGAGGTACCGAGGAATGCATTGCAGAAAGG	pLM08 construction
OL3250	GACTGACCCGGGTTGACAATGCTTGACTACGAA	pLM06 construction
OL3251	GACTGACCCGGGCCGCTAACCTTTGTGTTGTAC	pLM06 construction
OL3253	GACTGAGGCGCCAACCTCCCCTTGTCACTATTT	pLM09 construction
OL3254	GACTGAGGCGCCAATGATGTCTTCTCCGAAACC	pLM09 construction
OL3255	TACAGGTATCGCATTGTCAAG	*rec27* mutant library construction; *rec27* allele and *rec27-257::ura4*$^{+}$ identification
OL3256	TTTAGAAACGATTAGCCTCAC	*rec27* mutant library construction; *rec27* allele and *rec27-257::ura4*$^{+}$ identification
OL3257	ACCGCGAACATAGTCGGATTT	*rec25* mutant library construction; *rec25* allele identification
OL3258	AATGGGTTTAAAAGAATGCTG	*rec25* mutant library construction; *rec25* allele and *rec25-256::ura4*$^{+}$ identification
OL3259	GACGCGTAGACACTATTAACA	*mug20* mutant library construction; *mug20* allele identification
OL3260	CTTGGTTTGAGGCAGTATGAC	*mug20* mutant library construction; *mug20-*

		258::ura4^{+} identification
OL3266	AATGCGACCCTCATTTTAACTTATTAGTATTTGTAAAC GAATTTCAATCCAACCGCGAACATAGTCGGATTTAAAA ATGGATGCTAGAGTATTTCAAAGC	*rec25-256::ura4*$^{+}$ construction
OL3267	ATTAATAACATTTAGATGAAAAAGTAGTAGAGTTGGA ATAAATTTAGCTTTGAGTTTCAATCGTAATTTAGCTTAT TTAATGCTGAGAAAGTCTTTGCTG	*rec25-256::ura4*$^{+}$ construction
OL3268	AGTACGCTATATATCGCTAGCTTGATGTAAACAAAAG ACGCGTAGACACTATTAACATGTTATCAAGAACCACAA C ATGGATGCTAGAGTATTTCAAAGC	*mug20-258::ura4*$^{+}$ construction
OL3269	CGTGTTTGATGAAAGTTGACTTTCTTCGTTATTATCAA GCTTGGTTTGAGGCAGTATGACATTAGATCTCTGAAAT TTAATGCTGAGAAAGTCTTTGCTG	*mug20-258::ura4*$^{+}$ construction
OL3399	TCATACTTTTTGACGCGTTCTTCAATGTTTTTGAGGTTT AACTCTTCCAAAACA	*rec27-239* site-directed mutagenesis
OL3400	TGTTTTGGAAGAGTTAAACCTCAAAAACATTGAAGAA CGCGTCAAAAAGTATGA	*rec27-239* site-directed mutagenesis
OL3401	GGAAACATCCTTTATAAAAGCCTTTTCCTCATTTTCTAT ATTGGTCTTGTATT	*rec27-242* site-directed mutagenesis
OL3402	AATACAAGACCAATATAGAAAATGAGGAAAAGGCTTT TATAAAGGATGTTTCC	*rec27-242* site-directed mutagenesis
OL3403	ACCTGGGAAACATCCTCTATAAAAGCCTTTTTCTCATTT TCTATATTG	*rec27-243* site-directed mutagenesis
OL3404	CAATATAGAAAATGAGAAAAAGGCTTTTATAGAGGAT GTTTCCCAGGT	*rec27-243* site-directed mutagenesis
OL3405	CCGTTTTATTTTCGTACATTTCCTGTTGAACCTGGGAAA CATC	*rec27-244* site-directed mutagenesis
OL3406	GATGTTTCCCAGGTTCAACAGGAAATGTACGAAAATA AAACGG	*rec27-244* site-directed mutagenesis
OL3407	TTTTAACAACAATTCGCAGTTCCTCGGCATACTGTAAC AACTG	*rec27-245* site-directed mutagenesis
OL3408	CAGTTGTTACAGTATGCCGAGGAACTGCGAATTGTTG TTAAAA	*rec27-245* site-directed mutagenesis
OL3438	ATAGCCAGTGGGATTTGTAGCTAAGCTATGCTAAAAT ATACAACAGA	*rec27-257::ura4*$^{+}$ construction
OL3439	CAAAAAGTTTCGTCAATATCACAAGCTAGTCATTTTAA ACACATTAT	*rec27-257::ura4*$^{+}$ construction
OL3440	GAGATGAAAAGCTGCAGACT	*rec27-257::ura4*$^{+}$ construction
OL3441	GATCAATTCCAGAAGGCCAC	*rec27-257::ura4*$^{+}$ construction

OL3442	CAGACTTTCCTTCGCTCCAG	*rec27-257::ura4*$^{+}$ construction
OL3443	AGAAGGCCACAAATGGTAAGA	*rec27-257::ura4*$^{+}$ construction
OL3693	CAAAGTCCGACTCGTCTTCC	Probe amplification
OL3694	TAGAAGTGTAAGGCGCACGG	Probe amplification

Supplementary Table S3. Meiotic recombination of *rec25*, *rec27* and *mug20* mutants

Mutant	Gene conversion to *ade6*$^{+}$ [a]		Crossing-over (cM)[b] (*ade6 – arg1*)
	ade6-M26 × 3049	***ade6-M26 × 52***	
rec25^{+}	315 ± 20 (3)	18.7 ± 0.96 (4)	62 ± 6.8 (4)
rec25Δ	8.2 ± 1.3 (3)	0.34 ± 0.016 (4)	3.3 ± 0.4 (4)
rec25-234	7 ± 1 (4)	0.35 ± 0.027 (4)	2.2 ± 0.3 (4)
rec25-235	10.6 ± 1 (4)	0.4 ± 0.017 (4)	3.9 ± 0.8 (4)
rec25-236	8.9 ± 0.6 (4)	0.36 ± 0.023 (4)	3.3 ± 0.5 (4)
rec25-237	10.5 ± 0.2 (4)	0.39 ± 0.018 (4)	3.3 ± 0.7 (4)
rec27^{+}	293 ± 12 (12)	17.4 ± 0.8 (8)	55.8 ± 10.5 (4)
rec27Δ	12 ± 1 (12)	0.39 ± 0.02 (4)	2.9 ± 0.57 (4)
rec27-238	90 ± 3 (4)	10 ± 0.7 (5)	38.6 ± 3.8 (5)
rec27-239	39 ± 4 (4)	ND[c]	ND
rec27-240	42 ± 1.7 (4)	3.6 ± 0.2 (5)	20.8 ± 3 (5)
rec27-241	8.6 ± 0.3 (4)	0.33 ± 0.03 (4)	2.5 ± 0.68 (4)
rec27-242	273 ± 17 (4)	ND	ND
rec27-243	264 ± 11 (4)	ND	ND
rec27-244	260 ± 15 (4)	ND	ND
rec27-245	252 ± 8.4 (4)	ND	ND
rec27-246	11.5 ± 0.9 (4)	0.3	3
rec27-247	216 ± 6.6 (4)	12.58	55
rec27-248	183 ± 15 (4)	15.02	77
rec27-249	246 ± 6.4 (4)	16.24	51
mug20^{+}	290 ± 10 (12)	19.2 ± 1.16 (4)	58 ± 4.1 (4)
mug20Δ	10 ± 0.5 (8)	0.22 ± 0.02 (4)	3.8 ± 1.1 (4)
mug20-250	11 ± 1 (4)	0.17 ± 0.03 (4)	2.2 ± 0.7 (4)
mug20-251	130 ± 6.7 (4)	5.4 ± 0.35 (4)	38 ± 2.5 (4)

[a] Ade^{+} spores/10^{4} viable spores, as mean ± SEM from n crosses (in parentheses).

[b] 140 spore colonies from each of the four or five (as shown in parentheses) crosses were tested for *ade6 – arg1* recombinants. Frequencies were converted to cM using Haldane's equation, as mean ± SEM.

[c] ND, not determined.

References

1 Larkin, M. A. *et al.* Clustal W and Clustal X version 2.0. *Bioinformatics* **23**, 2947-2948 (2007).

2 Helston, R. M., Box, J. A., Tang, W. & Baumann, P. Schizosaccharomyces cryophilus sp. nov., a new species of fission yeast. *FEMS Yeast Res.* **10**, 779-786 (2010).

3 DeVeaux, L. C., Hoagland, N. A. & Smith, G. R. Seventeen complementation groups of mutations decreasing meiotic recombination in *Schizosaccharomyces pombe*. *Genetics* **130**, 251-262 (1992).

4 Ponticelli, A. S. & Smith, G. R. Meiotic recombination-deficient mutants of *Schizosaccharomyces pombe*. *Genetics* **123**, 45-54 (1989).

5 Davis, L., Rozalén, A. E., Moreno, S., Smith, G. R. & Martin-Castellanos, C. Rec25 and Rec27, novel components of meiotic linear elements, link cohesin to DNA breakage and recombination in fission yeast. *Curr. Biol.* **18**, 849-854 (2008).

6 Lorenz, A. *et al. S. pombe* meiotic linear elements contain proteins related to synaptonemal complex components. *J. Cell Sci* **117**, 3343-3351 (2004).

7 Steiner, W. W. & Smith, G. R. Optimizing the nucleotide sequence of a meiotic recombination hotspot in *Schizosaccharomyces pombe*. *Genetics* **169**, 1973-1983 (2005).

8 Schuchert, P., Langsford, M., Kaslin, E. & Kohli, J. A specific DNA sequence is required for high frequency of recombination in the ade6 gene of fission yeast. *EMBO J.* **10**, 2157-2163 (1991).

9 Estreicher, A., Lorenz, A. & Loidl, J. Mug20, a novel protein associated with linear elements in fission yeast meiosis. *Curr. Genetics* **58**, 119-127, doi:10.1007/s00294-012-0369-3 (2012).

10 Iino, Y. & Yamamoto, M. Mutants of *Schizosaccharomyces pombe* which sporulate in the haploid state. *Mol. and General Genet.* **198**, 416-421 (1985).

11 Farah, J. A., Hartsuiker, E., Mizuno, K.-I., Ohta, K. & Smith, G. R. A 160-bp palindrome is a Rad50•Rad32-dependent mitotic recombination hotspot in *Schizosacchromyces. pombe*. *Genetics* **161**, 461-468 (2002).

12 Pryce, D. W., Lorenz, A., Smirnova, J. B., Loidl, J. & McFarlane, R. J. Differential activation of M26-containing meiotic recombination hot spots in Schizosaccharomyces pombe. *Genetics* **170**, 95-106, doi:10.1534/genetics.104.036301 (2005).

13 Ellermeier, C. & Smith, G. R. Cohesins are required for meiotic DNA breakage and recombination in *Schizosaccharomyces pombe*. *Proc. Natl. Acad. Sci. USA* **102**, 10952-10957 (2005).

14 Martin-Castellanos, C. *et al.* A large-scale screen in *S. pombe* identifies seven novel genes required for critical meiotic events. *Curr. Biol.* **22**, 2056-2062 (2005).
